# NupR Responding to Multiple Signals Is a Nucleoside Permease Regulator in Bacillus thuringiensis BMB171

**DOI:** 10.1128/spectrum.01543-22

**Published:** 2022-07-07

**Authors:** Jiaxin Qin, Zhanglei Cao, Xia Cai, Yu Fang, Baoju An, Xuelian Li, Yizhuo Zhang, Hongwei Tian, Wenzhuo Hu, Bing Yan, Jun Cai

**Affiliations:** a Department of Microbiology, College of Life Sciences, Nankai University, Tianjin, China; b Key Laboratory of Molecular Microbiology and Technology, Ministry of Education, Tianjin, China; c Tianjin Key Laboratory of Microbial Functional Genomics, Tianjin, China; Griffith University

**Keywords:** GntR, NagR-like proteins, nucleoside transporter, nucleoside utilization, riboswitch, guanine

## Abstract

Nucleoside transport is essential for maintaining intracellular nucleoside and nucleobase homeostasis for living cells. Here, we identified an uncharacterized GntR/HutC family transcriptional regulator, NagR2, renamed NupR (nucleoside permease regulator), that mainly controls nucleoside transport in the Bacillus thuringiensis BMB171 strain. The deletion or overexpression of *nupR* affected the bacteria's utilization of guanosine, adenosine, uridine, and cytidine rather than thymidine. We further demonstrated that zinc ion is an effector for the NupR, dissociating NupR from its target DNA. Moreover, the expression of *nupR* is inhibited by NupR, ComK, and PurR, while it is promoted by CcpA. Also, a purine riboswitch located in its 5′ noncoding region influences the expression of *nupR*. Guanine is the ligand of the riboswitch, reducing the expression of *nupR* by terminating the transcription of *nupR* in advance. Hence, our results reveal an exquisite regulation mechanism enabling NupR to respond to multiple signals, control genes involved in nucleoside transport, and contribute to nucleoside substance utilization. Overall, this study provides essential clues for future studies exploring the function of the NupR homolog in other bacteria, such as Bacillus cereus, Bacillus anthracis, Klebsiella pneumoniae, and Streptococcus pneumoniae.

**IMPORTANCE** The transport of nucleosides and their homeostasis within the cell are essential for growth and proliferation. Here, we have identified a novel transcription factor, NupR, which, to our knowledge, is the first GntR family transcription factor primarily involved in the regulation of nucleoside transport. Moreover, responding to diverse intracellular signals, NupR regulates nucleoside transport. It is vital for utilizing extracellular nucleosides and maintaining intracellular nucleoside homeostasis. NupR may also be involved in other pathways such as pH homeostasis, molybdenum cofactor biosynthesis, nitrate metabolism, and transport. In addition, nucleosides have various applications, such as antiviral drugs. Thus, the elucidation of the transport mechanism of nucleosides could be helpful for the construction of engineered strains for nucleoside production.

## INTRODUCTION

In coping with complex and changeable environments, bacteria utilize various regulatory systems, especially transcriptional regulation, to tightly modulate the expression of genes (1). Transcription factors binding to DNA promoters to regulate the transcription of downstream genes are the most common control mode, which is well documented (2). However, the functions of many valuable transcription factors are undiscovered.

NagR (once named YovA) is one of the typical transcriptional regulators in the GntR/HutC family, the most widely distributed helix-turn-helix (HTH) transcriptional regulator in bacteria. In Bacillus subtilis, YovA/NagR is a repressor that serves as a negative transcriptional regulator in *N*-acetylglucosamine (GlcNAc) transport and utilization. Its effector in B. subtilis is glucosamine-6-phosphate (GlcN-6-P) or *N*-acetylglucosamine-6-phosphate (GlcNAc-6-P), which could abolish its binding to the target gene (2, 3). In Bacillus thuringiensis, besides GlcNAc transport and utilization, NagR regulates the expression of chitinase and chitin-binding protein involved in the chitinase degradation (4). In addition, GlcNAc is an effector of NagR in B. thuringiensis, which is in agreement with NagR in B. subtilis (5).

**FIG 1:**
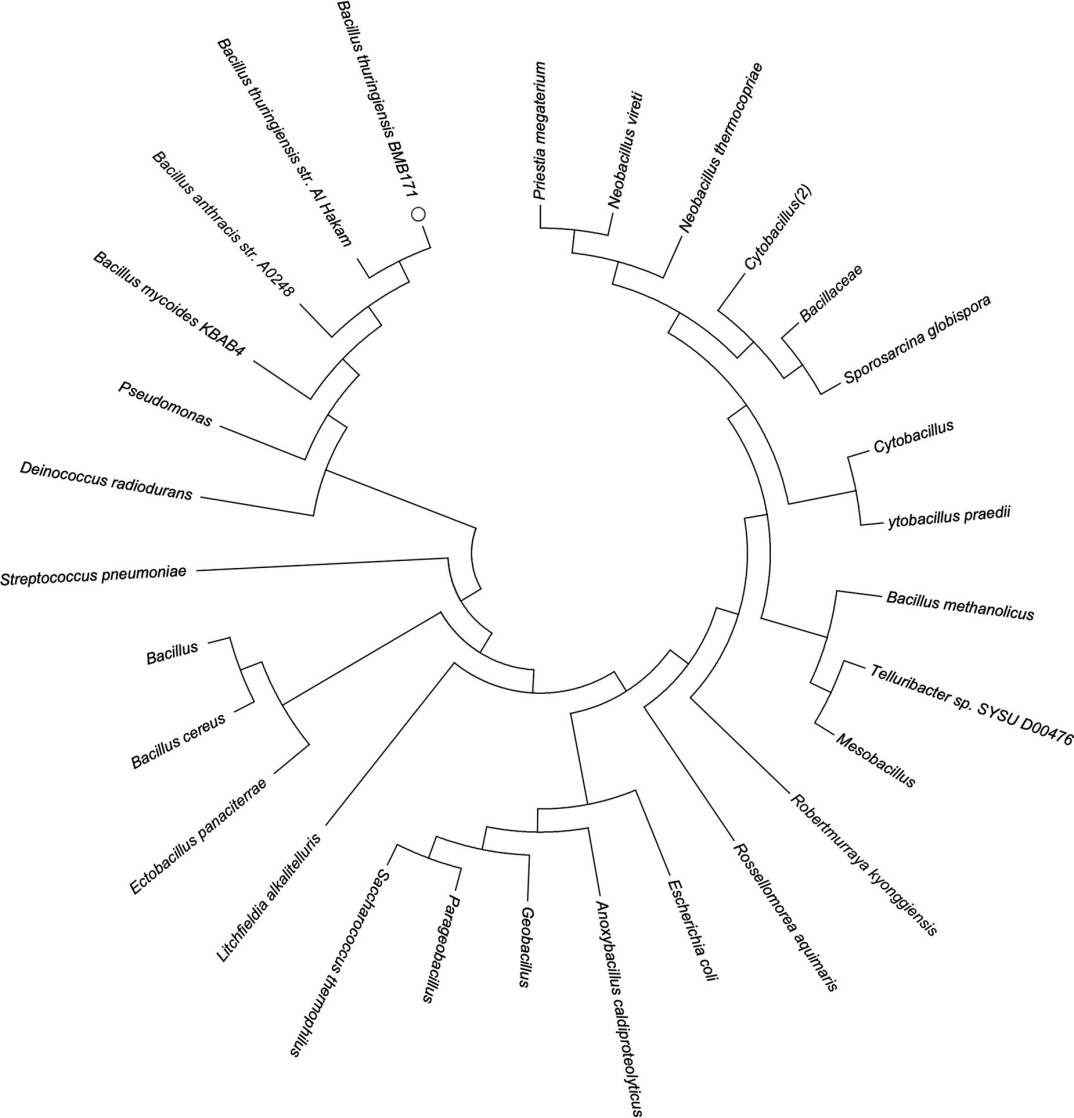
Phylogenetic tree of NagR2 homologs. Red circle marks NagR2 of BMB171 strain.

By homology comparison, we found the other GntR/HutC transcription factor, NagR2, in BMB171. The identity between NagR and NagR2 is only 24.21%. Furthermore, the NagR2 similar proteins are widely present in other bacteria, such as Bacillus subtilis, Bacillus anthracis, Clostridioides difficile, Pseudomonas aeruginosa, and Streptococcus pneumoniae (Fig. 1). However, the function of NagR2 remains undefined. Interestingly, we discovered that a predicted purine riboswitch resided in its 5′ untranslated region (5′ UTR). It is a rare phenomenon that riboswitch tunes the expression of transcriptional factors. Therefore, we wondered whether the predicted riboswitch modulates the expression of NagR2.

In the present study, we investigated the function of NagR2 and explored the transcriptional regulation mechanism of the expression of *nagR2*.

## RESULTS

### Transcriptome analysis.

**FIG 2:**
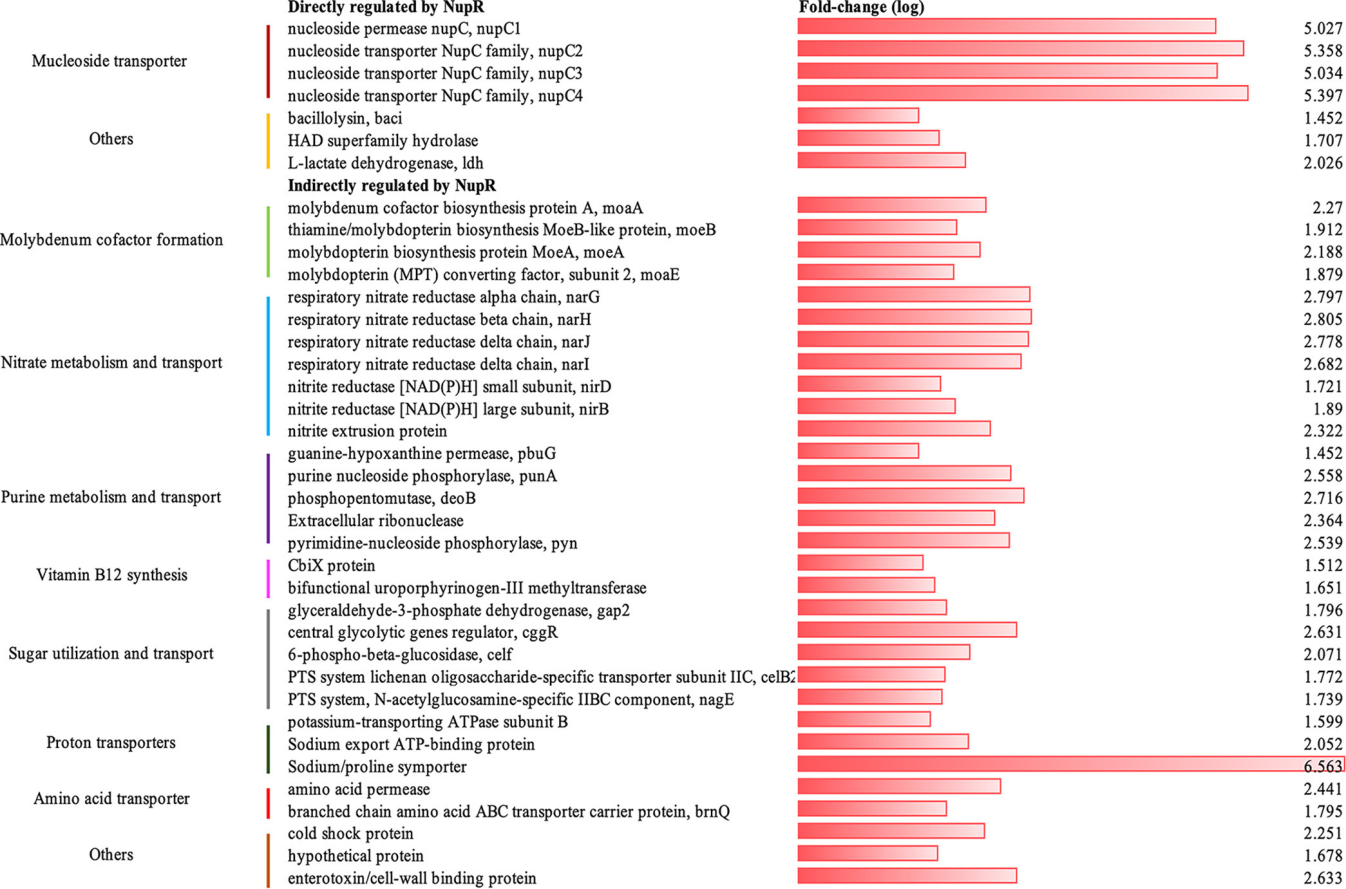
Fold change of upregulated genes in △*nagR2*.

To explore the regulatory network of NagR2, we used Solexa/Illumina sequencing to perform a transcriptome sequencing analysis for both BMB171 and BMB171(△*nagR2*) in the exponential phase (~9 h). To verify the reliability of the sequencing results, we performed reverse transcription-quantitative PCR (qRT-PCR) with 10 differentially expressed genes (DEGs) selected randomly. As shown in Fig. S1 in the supplemental material, although there were quantitative differences, the results of the qRT-PCR analysis were consistent with those of the sequencing analysis. Of the 123 differentially expressed genes, the expression of 38 genes is at higher levels (Fig. 2) and 85 genes at lower levels in the Δ*nagR2* strain (*P* < 0.05) (see Table S1 in the supplemental material). The transcriptome sequencing (RNA-seq) data were deposited at NCBI BioProject (accession no. PRJNA825124).

**FIG 3:**
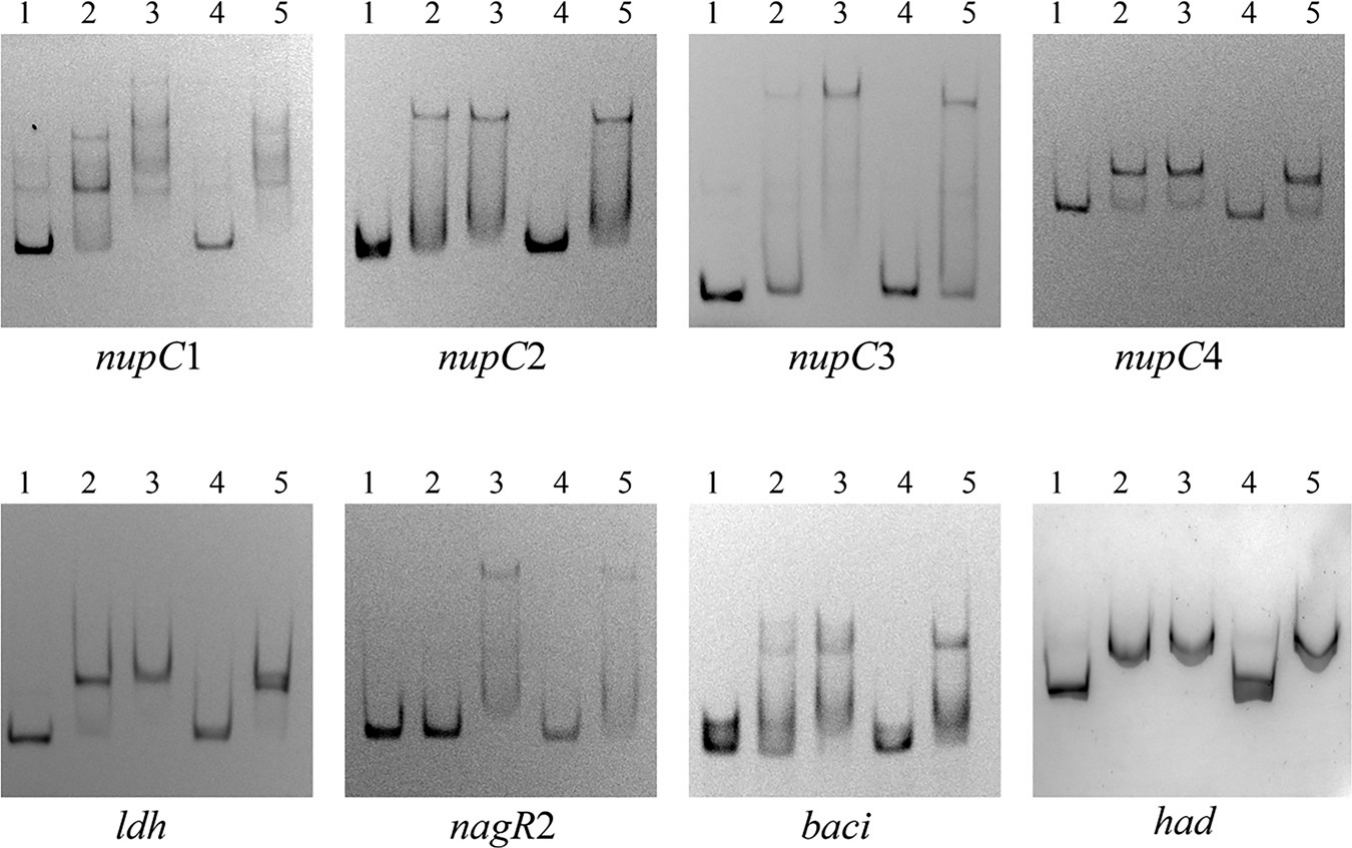
NagR2 binds directly to the promoter regions of the eight genes. In lanes 1 to 3, increasing amounts of the purified NagR2-his protein (0, 0.05, and 0.1 μmol/L) were incubated with the 0.2 μmol/L Cy5-labeled promoter region of *nupC1*, *nupC2*, *nupC3*, *nupC4*, *ldh*, *nagR2*, *baci*, and *bad*, respectively. Lane 4 shows a specific competitor with 0.2 μmol/L DNA, 0.1 μmol/L NagR2 protein, and 400× unlabeled competitive DNA, and lane 5 shows a noncompetitor with 0.2 μmol/L DNA, 0.1 μmol/L NagR2 protein, and 0.5 μg/μL salmon sperm DNA.

Due to a large amount of DEGs given by transcriptome sequencing, 84 genes with significant differences, explicit annotation, and essential biological functions were selected for electrophoretic mobility shift assay (EMSA) experimental validation listed in Table S1. With EMSA verification, band shifts were observed with the promoter regions of *nupC1*, *nupC2*, *nupC3*, *nupC4*, *ldh*, *baci*, *had*, and *nagR2* (Fig. 3). The first four genes are annotated as nucleoside permease genes involved in nucleoside transport. Transcription factors are generally self-regulated, and NagR2 is no exception and can bind to its promoter region. Moreover, we verified that *nagR2* and four genes coding for nucleoside transporter-associated proteins located downstream were cotranscribed and comprised an operon, which we named *nup* operon. Transcriptional activity of the *nagR2* promoter in the Δ*nagR2* strain is significantly higher than that in BMB171 based on the β-galactosidase activity test (Fig. S2). Hence, NagR2 directly inhibits the expression of the *nup* operon. To sum up the above, NagR2 directly modulates the expression of eight genes encoding nucleoside transporter proteins. Thus, we renamed NagR2 Nucleoside permease Regulator (NupR). The rest of the three genes directly regulated by NupR, respectively, encode bacillolysin (BMB171_RS26735, *baci*), HAD hydrolase superfamily (BMB171_C4990, *had*), and l-lactate dehydrogenase (BMB171_RS09485, *ldh*) (Fig. 3).

The promoters of the 31 upregulated differentially expressed genes NupR cannot bind are listed below. Deletion of *nupR* may be conducive to the formation of molybdenum cofactor formation. Four genes, *moaA* (BMB171_RS10525), *moeB* (BMB171_RS10530), *moeA* (BMB171_RS10535), and *moaE* (BMB171_RS10540), are in an operon and participate in the biosynthesis of molybdenum cofactor (Moco) (6). In prokaryotes, the maturation of pterin-type molybdenum cofactors includes nucleotide modifications. Therefore, the expression of genes related to molybdenum cofactor synthesis may be affected by intracellular nucleoside concentrations, which could be affected by NupR (7). Also, nitrate metabolism and transport may change in Δ*nupR*. The expression of α-chain respiratory nitrate reductase (BMB171_RS10500, *narG*), β-chain respiratory nitrate reductase (BMB171_RS10505, *narH*), δ-chain respiratory nitrate reductase (BMB171_RS10510, *narJ*), γ-chain respiratory nitrate reductase (BMB171_RS10515, *NarI*), small subunit nitrite reductase [NAD(P)H] (BMB171_RS10585), significant subunit nitrite reductase [NAD(P)H] (BMB171_RS10590), and nitrite extrusion protein (BMB171_RS10550) are upregulated, which could be caused by the acceleration of molybdenum fixation in nitrogenase and nitrate reductase (8).

In addition, purine metabolism and transport are influenced by the deletion of NupR. The change in the expression of the following genes, *pbuG* (BMB171_RS20290), *pyn* (BMB171_RS20300), *punA* (BMB171_RS20305), *deoB* (BMB171_RS20310), and BMB171_RS16385 coding for extracellular RNase (9–12), may be caused by the alteration of nucleoside transport in Δ*nupR*. Also, vitamin B_12_ participates in DNA, fatty acid, and amino acid synthesis. In the Δ*nupR* strain, its production may increase due to increased expression of genes coding for cobalamin biosynthesis protein (BMB171_RS10575) and bifunctional uroporphyrinogen methyltransferase (BMB171_RS10580).

The absence of *nupR* also indirectly affects sugar utilization and transport. The expression of *cggR* (BMB171_RS25590, central glycolytic genes regulator), *gap2* (BMB171_RS25585, glyceraldehyde-3-phosphate dehydrogenase) modulated by CggR (13), *celf* (BMB171_RS25960, 6-phospho-beta-glucosidase), *nagE* (BMB171_RS02520, *N*-acetylglucosamine-specific IIBC component), and *celB2* (BMB171_RS25970, lichenan oligosaccharide-specific transporter subunit) also increases in Δ*nupR*.

Moreover, deletion of NupR may affect the pH homeostasis of BMB171. In △*nupR* strain, the expression of *ldh* is upregulated, and lactate production may be affected, resulting in an imbalance of cell pH homeostasis (14). Genes coding for proton transporters, such as BMB171_RS03885, BMB171_RS06420, and BMB171_RS18055, are upregulated in Δ*nupR*. The high expression of the proton transporters could help maintain intracellular pH homeostasis.

Furthermore, EMSA results indicated that no band shifts appeared with the promoter regions of the 85 downregulated DEGs (Table S1). We will not dwell on it here.

### NupR-binding sites in the BMB171 genome.

**FIG 4:**
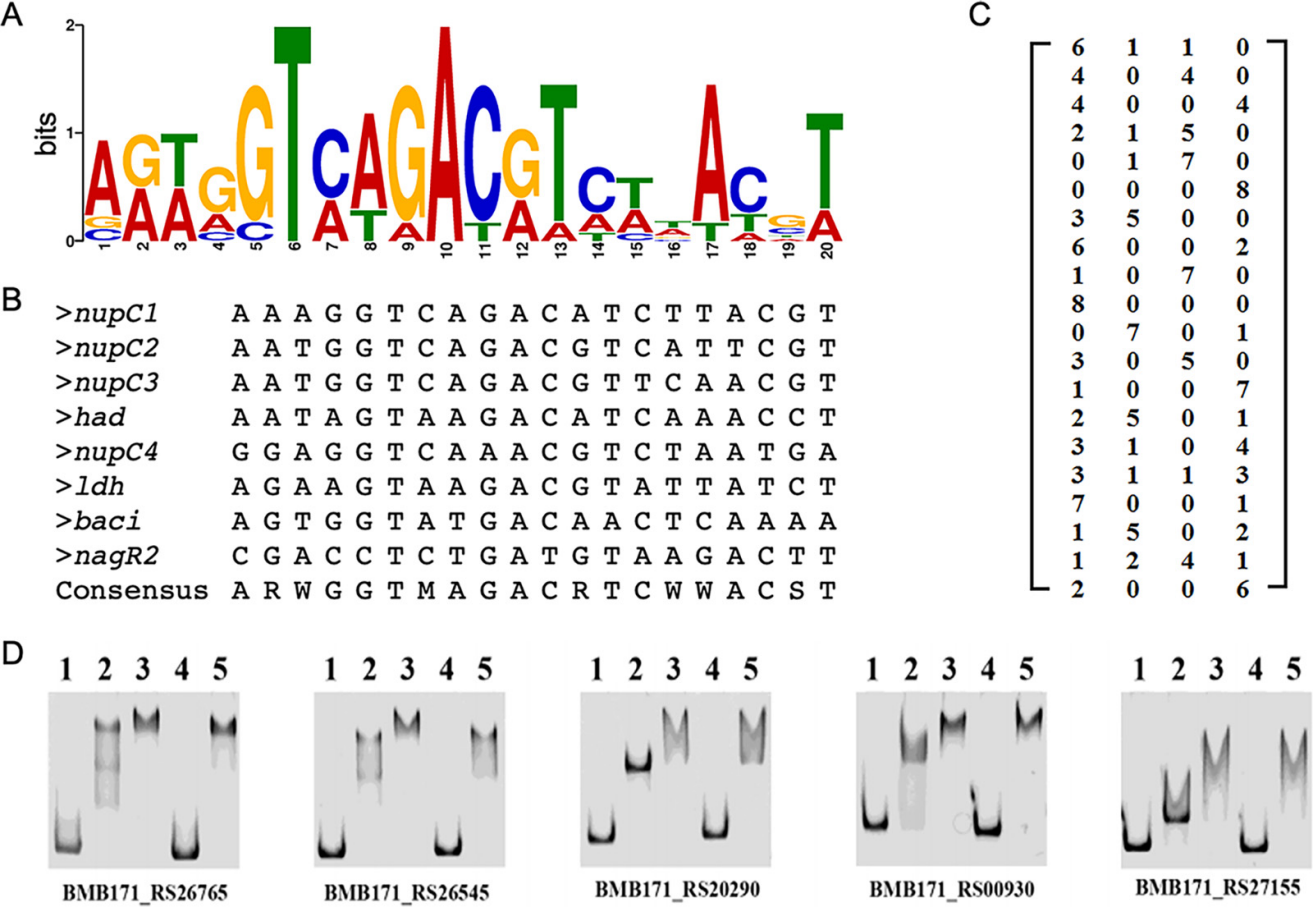
Identification of potential NupR binding motif. (A) Potential NupR binding motif analyzed by MEME using the eight DNA sequences bound by NupR in Fig. 3. (B) Sequence logo representation generated by the WebLogo tool. (C) Position weighting matrix (PWM) for the NupR binding motif. (D) EMSA for detecting protein-DNA interactions using five Cy5-labeled DNA sequences and increasing concentrations of recombinant NupR-His. The five additional DNA sequences were found in the whole BMB171 genome by FIMO software using the PWM in panel C. Lanes 1 to 2 contain 0 and 0.05 μmol/L NupR-His, respectively, and lanes 3 to 5 contain 0.1 μmol/L. Lane 4 contains 400× unlabeled competitive DNA, and lane 5 contains 0.5 μg/μL salmon sperm DNA.

Based on the eight DNA sequences binding with NupR, MEME analysis revealed a potential NupR-binding site, as shown in Fig. 4A and B. Although the 20-bp consensus sequence is not a perfect palindrome sequence, the sixth and tenth bases are the most conserved, conforming to the DNA binding sequence of the GntR family. We named this consensus sequence the *nup* site.

The *nup* site recognition matrix is shown in Fig. 4C. Using the matrix, we scanned the binding sites in the whole genome of BMB71 by FIMO software. Furthermore, five more *nup* binding sites were verified by EMSA (Fig. 4D). The genetic locus tag, fold change, and *nup* sequence of the 13 genes are listed in Table 1. The binding site of NupR can be not only in the promoter region but also in the coding region. It has also been reported that typical CcpA-binding sites were within promoters or coding regions (15). However, further experimental verification is needed to determine whether the binding site in the latter has biological significance.

**TABLE 1:** Genes directly regulated by NagR2

Genetic locus tag	Description	Fold change	*Nup* sequence	Position
BMB171_RS01930	Nucleoside permease	5.027	AAAGGTCAGACATCTTACGT	Pro^*a*^
BMB171_RS09485	L-Lactate dehydrogenase	2.026	AGAAGTAAGACGTATTATCT	Pro
BMB171_RS25245	Nucleoside transporter NupC family	5.358	AATGGTCAGACGTCATTCGT	Pro
BMB171_RS25250	Nucleoside transporter NupC family	5.034	AATGGTCAGACGTTCAACGT	Pro
BMB171_RS26110	Nucleoside transporter NupC family	5.397	GGAGGTCAAACGTCTAATGA	Pro
BMB171_RS26735	Bacillolysin	1.452	AGTGGTATGACAACTCAAAA	Pro
BMB171_RS26950	HAD superfamily hydrolase	1.707	AATAGTAAGACATCAAACCT	Pro
BMB171_RS18795	GntR family transcriptional regulator		CGACCTCTGATGTAAGACTT	Pro
BMB171_RS26765	Collagen adhesion protein	5.91	AAAGGTCTGATATCATTCTT	Pro
BMB171_RS26545	Uracil phosphoribosyltransferase	6.51	CGAAGTAAGACAACTGACGA	Pro
BMB171_RS20290	Uracil permease	7.54	ACAGGTCTGACGTCTATCGT	CDS^*b*^
BMB171_RS00930	Diadenylate cyclase	2.39	ATTGGTAAGATGTCAAACAT	Pro
BMB171_RS27155	Hypothetical protein	7.48	AGTGGTAAGACCTATTAAAA	Pro

a Promoter.

b Coding sequence.

### Effect of NupR on nucleoside transport.

**FIG 5:**
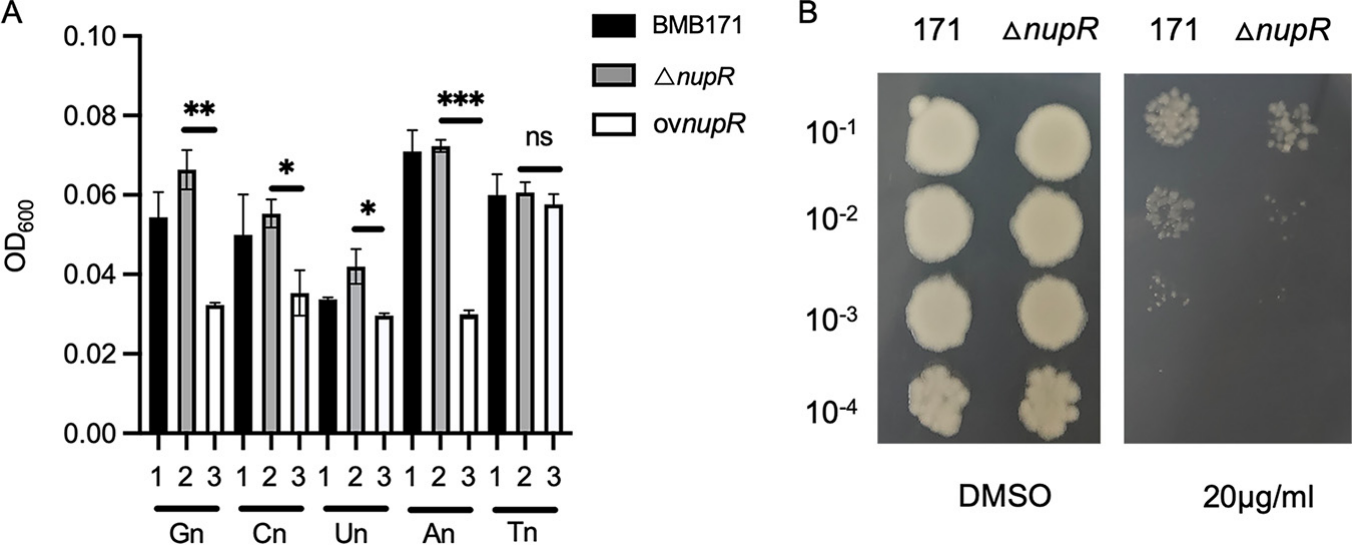
NupR impacts the utilization of nucleosides. (A) OD_600_ values at the 4th of BMB171, △*nupR*, and overexpression *nupR* strains in M9 medium with 1 mM different nucleosides. Gn, guanosine; Cn, cytidine; Un, uridine; An, adenosine; Tn, thymidine. ns, not significant; *, *P < *0.05; **, *P < *0.01; ***, *P < *0.001 by Student's *t* test. Each data point represents the mean value from at least three independent replicates. Error bars show the standard errors of the means. (B) Growth of BMB171 and △*nupR* strains on LB solid media containing 20 μg/mL 5-fluorouridine. DMSO, dimethyl sulfoxide. Data represent results from three independent experiments.

Since NupC1, NupC2, NupC3, and NupC4 have been annotated as nucleoside permeases involved in nucleoside transport, we examined the role of NupR in nucleoside utilization. The growth curves of wild-type strain BMB171, *nupR* knockout (△*nagR2*/*nupR*), and *nupR* overexpression strains in the M9 medium containing different nucleosides were determined. At the 4th hour, the optical density at 600 nm (OD_600_) values of BMB171 and *nupR* knockout were not noticeably different when diverse nucleosides were added. Nevertheless, *nupR* knockout and *nupR* overexpression strains had a visible difference with the addition of 1 mM guanosine, adenosine, uridine, or cytidine instead of thymidine (Fig. 5A). We inferred that although *nupR* exists in the BMB171 strain, the riboswitch or transcription factors may inhibit its transcriptional activity. Hence, nucleoside transporters regulated negatively by NupR could generally transport different nucleosides at a certain level. In the *nupR* overexpression strain, the expression of *nupR* increases and inhibits the expression of nucleoside transporters. The results suggested that nucleoside transporters governed by NupR could transport guanosine, adenosine, cytidine, or uridine rather than thymidine.

We also determined the effect of 5-fluorouridine on the growth of wild-type and mutant strains on LB solid medium. First, wild-type BMB171 and Δ*nupR* strains were grown in LB medium. At the middle of the logarithm, 1 mL bacterial solution was taken, diluted, and dropped into LB solid medium with or without 5-fluorouridine. Figure 5B shows that both the wild-type and knockout strains grew well, and there were no differences between them on LB solid medium without 5-fluorouridine. However, in the presence of 20 μg/mL of 5-fluorouridine, the growth of the wild-type strain was greater than that of the knockout strain. Therefore, the results suggested that the deletion of *nupR* could upregulate the expression of nucleoside transporters and lead to the import of 5-fluorouridine. As a result, the more 5-fluorouridine enters the cells, the worse cells grow. It is consistent with the growth curve results that the overexpression of *nupR* could affect bacteria's uridine utilization.

### Zinc ion is the ligand of NupR.

The regulatory range of NupR is significantly different from that of the NagR (YovA). SWISS-MODEL analysis was used to search the protein templates using the NupR amino acid sequence. The predicted structure of the NupR protein mostly resembles that of NagR (formerly named YvoA), an HTH-type transcriptional repressor that belongs to the GntR family from B. subtilis. Fillenberg et al. reported that NagR in B. subtilis mainly controls the uptake and metabolism of GlcNAc (2). The crystal structure of NagR is complex with the putative effector molecules GlcN-6-P or GlcNAc-6-P. However, in BMB171, NupR primarily regulates nucleoside transport. Therefore, we speculated that the ligand of NupR may be a nucleoside. To verify this hypothesis, we performed EMSAs to probe which nucleoside can hinder the binding between NupR and its target promoter DNA. However, all the tested nucleosides and nucleobases failed to affect the binding (data not shown).

**FIG 6:**
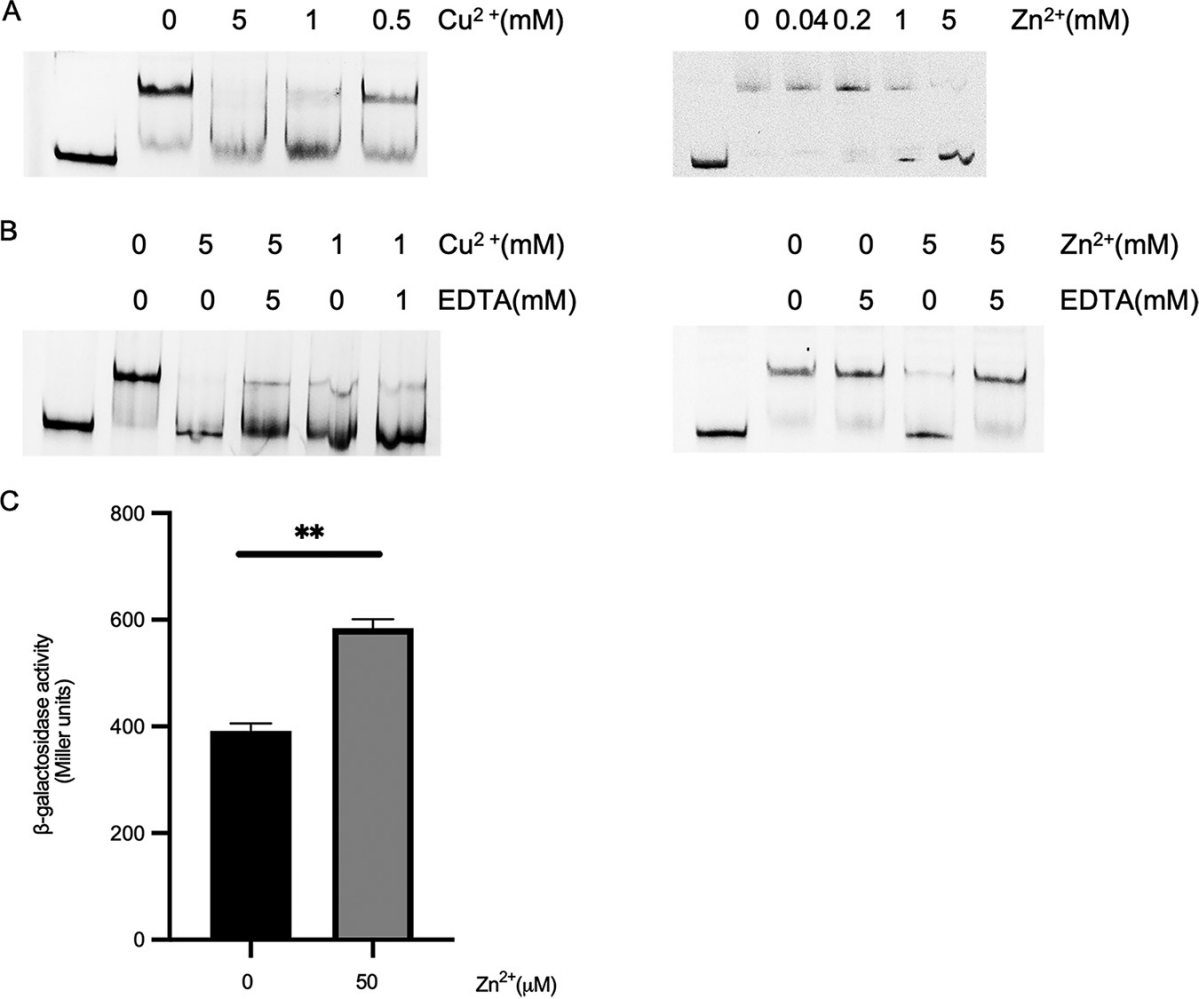
Influence of Zn^2+^ or Cu^2+^ on the activity of NupR protein *in vitro* (DNA binding) and the activity of the *nupR*-p14 promoter *in vivo*. (A) NupR lost DNA binding ability after adding 5 mM divalent metal ions Cu^2+^ or Zn^2+^. Increasing concentrations of Cu^2 +^ or Zn^2+^ were added to the mixture containing 0.2 μmol/L Cy5-labeled promoter region of *nupR*-p14 and 0.1 μmol/L NupR protein. (B) Cu^2+^, rather than Zn^2+^, denatures proteins. EMSA with 0.2 μmol/L labeled fragment *nupR*-p14, 0.1 μmol/L NupR protein, Zn^2+^ or Cu^2+^, and EDTA were added as indicated. (C) The activity of the *nupR*-p14 promoter in BMB171 strain was measured at the end of logarithmic-phase growth on SSM medium with Zn^2+^ at 50 μM. **, *P < *0.01 by Student's *t* test; each data point represents the mean value from at least three independent replicates. Error bars show the standard errors of the means.

A GntR-like transcription factor HypR can bind zinc ions. Hence, we tested the influence of several divalent metal ions on the binding between NupR and DNA (16). The EMSA analysis showed that NupR lost its DNA binding ability after adding divalent metal ions Cu^2+^ or Zn^2+^. Moreover, with increasing Cu^2+^ or Zn^2+^ concentration, the free DNA probe was apparent gradually, and almost no shift band when the concentration of Cu^2+^ or Zn^2+^ was up to 5 mM (Fig. 6A).

Furthermore, we performed the following experiments to exclude the possibility of denaturation of proteins by metal ions. EDTA was added to chelate the metal ions in the reaction after 15 min of reaction between protein and metal ions. Proteins denatured by metals cannot be renatured. If a protein can still bind to DNA after removing metal ions from the solution, the metal ion is not the denaturant for it. Figure 6B shows that the protein-DNA binding was restored after chelating the zinc ion but not copper, indicating that NupR may be denatured by the copper rather than the zinc ion. The results suggested that zinc ion may be the ligand of NupR.

If Zn^2+^ has a signaling function and its binding causes dissociation of the NupR protein from its target DNA sequences, the transcriptional activity of the genes regulated by NupR should increase in the presence of Zn^2+^. NupR represses the expression of itself. Therefore, we measured the transcriptional activity of *nupR* supplemented with ~0 to 50 μM Zn^2+^ in Schaeffer’s sporulation medium (SSM). In the strain BMB171(pHT1K-nupR-p14), the transcriptional activity of *nupR* determined by β-galactosidase increased gradually with increasing zinc ion concentration. In the presence of 50 μM Zn^2+^, the transcriptional activity of *nupR* was significantly higher than that without Zn^2+^(Fig. 6C). Instead, the transcriptional activity of *nupR* is not affected by the copper ions in SSM medium (Fig. S3). These results suggested that Zn^2+^ is the likely ligand for NupR, dissociating NupR from its target DNA and activating the gene expression repressed by NupR.

### Regulation of *nupR* by other transcription factors.

To investigate how *nupR* expression is regulated, except for NupR, we first analyzed the promoter region of *nupR* using the DBTBS database (https://dbtbs.hgc.jp) and riboswitch prediction software Rfam (http://rfam.xfam.org/). We noticed putative binding sites for the transcription factors ComK, CcpA, and PurR, and a hypothetical riboswitch regulatory element existed in the upstream region of the *nupR* gene.

**FIG 7:**
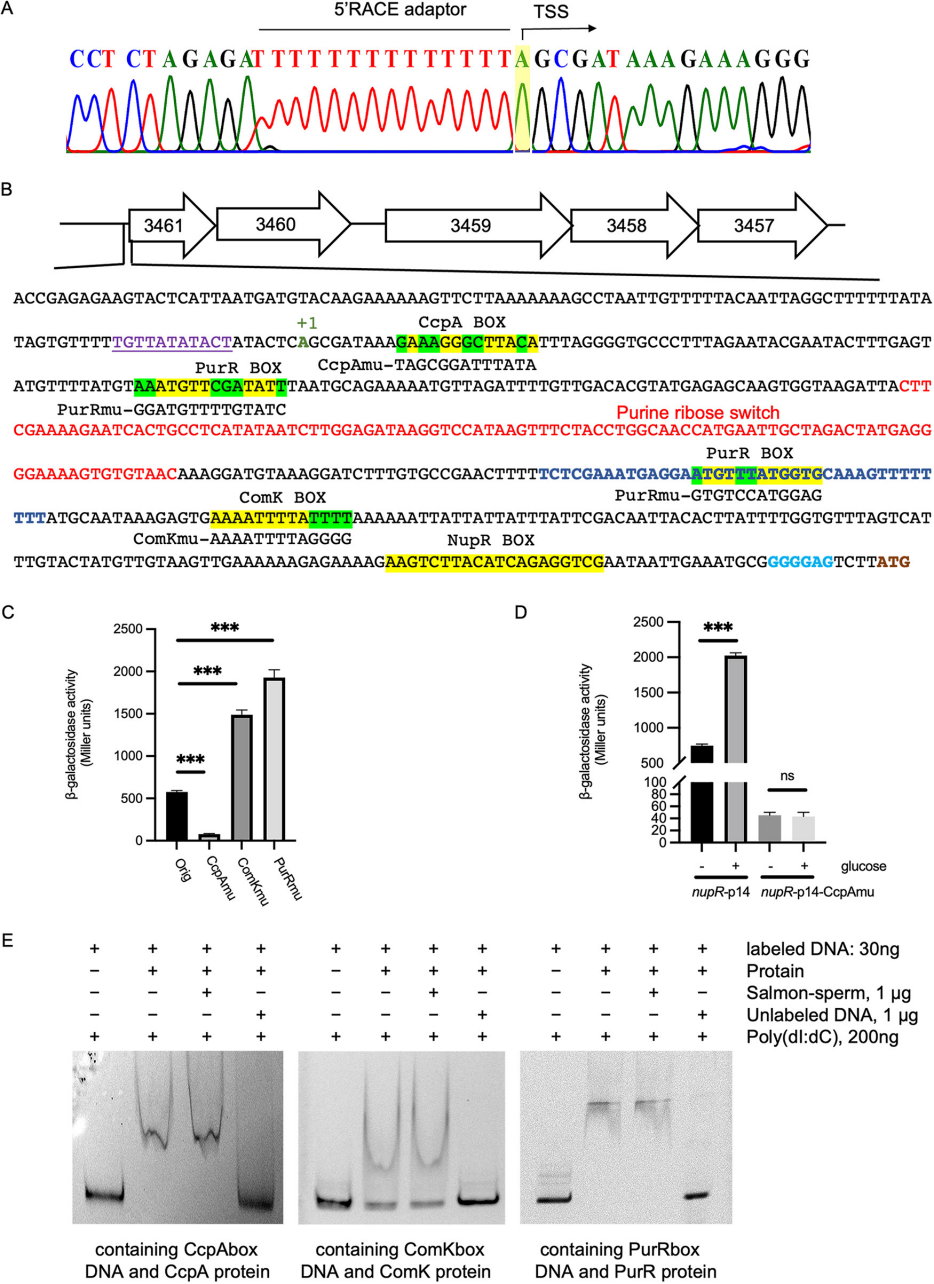
Promoter region of the *nup* operon and regulation of other transcription factors on *nup.* (A) The transcription start site (TSS) of *nup* was identified using 5′ RACE. The sequence of the 5′-RACE adaptor is indicated, and the TSS is shaded in yellow. (B) *nup* operon and the sequence of the *nup* promoter. The extended −10 promoter is marked in purple, and the binding sites of the transcription factors CcpA, PurR, ComK, and NupR are shaded in yellow. The conservative bases of transcription factor binding sites that need to be mutated are marked with a green shadow, and the mutated bases are shown under it. The TSS of *nupR*, purine ribose switch, the predicted intrinsic terminator, Shine-Dalgarno (SD), and the start codon of NupR are marked in green, red, deep blue, blue, and brown, respectively. 3461 to 3457, BMB171_C3461 (NupR/NagR), BMB171_C3460 (nucleoside-binding protein), BMB171_C3459 (nucleoside transport ATP-binding protein), and BMB171_C3458-3457 (nucleoside transport system permease protein), respectively. (C) Effects of the mutants in transcription factor CcpA/ComK/PurR conservative binding site on the transcription activity of pHT1K-*nupR*-p14. The β-galactosidase activity was assessed in the BMB171 strain containing the pHT1K-*nupR*-p14 (marked as Orig), pHT1K-*nupR*-p14-CcpAmu (marked as CcpAmu), pHT1K-*nupR*-p14-ComKmu (marked as ComKmu), and pHT1K-*nupR*-p14-PurRmu (marked as PurRmu). ***, *P < *0.001 by Student's *t* test; each data point represents the mean value from at least three independent replicates. Error bars show the standard errors of the means. (D) Glucose induces the expression of pHT1K-*nupR*-p14 and pHT1K-*nupR*-p14-CcpAmu. The β-galactosidase activity of pHT1K-*nupR*-p14 and pHT1K-*nupR*-p14-CcpAmu was detected in BMB171 strain with or without 0.1% glucose. ns, not significant; ***, *P < *0.001 by Student's *t* test. Data represent the mean ± SD from three independent replicates. (E) Interaction between CcpA/ComK/PurR and the corresponding DNA sequence containing the binding box. Electrophoretic mobility shift assay (EMSA) for detecting protein-DNA interactions using 30 ng corresponding labeled DNA fragment and the proteins CcpA (3.6 μg), ComK (20 ng), and PurR (40 ng). We added 200 ng poly(dI-dC) to inhibit nonspecific binding. Salmon sperm DNA was added for nonspecific competition. Unlabeled DNA was added for specific competition.

We performed 5′ rapid amplification of cDNA ends (RACE) to determine the transcription start site (TSS) of *nupR*. The defined TSS was located at the base “A,” 473 nucleotides (nt) upstream of the translation initiation site (TIS) of NupR (Fig. 7A). Based on that, −35 (TTTATA) and −10 (TTATATACT) regions were predicted by Softberry. The spacer region length between −10 and −35 regions is just 11 nucleotides, and −35 is too close to the −10 region to be functional. The predicted −10 region is preceded by “TG” and contains short runs of T residues in the spacer region, which is consistent with the extended −10 promoter. It has been reported that the extended −10 5′-TG-3′ element contributes to promoter activity, and its presence eliminates the dependence on the −35 region for some promoters (17). Thus, the promoter is shown in Fig. 7B.

The following research was performed to verify whether CcpA, PurR, or ComK transcription factor regulates the expression of *nupR*. First, several expression vectors of the *nupR* promoter fusion *lacZ* containing mutations in each transcription factor conservative binding site were constructed, respectively (Fig. 7B), and the activity of the promoters was measured. As shown in Fig. 7C, the transcriptional activity of *nupR*-p14mu*ccpA* was significantly lower than that of *nupR*-p14. Conversely, the transcriptional activity of *nupR*-p14 dramatically increased when a few bases in the putative binding box for ComK or PurR were mutated.

In the presence of a preferred carbon source such as glucose, CcpA could bind to the catabolite responsive element (*cre*) site of the target genes and block the utilization of secondary carbon sources (18). If CcpA could upregulate the expression of *nupR*, the transcription activity of *nupR* may be affected by organic carbon sources such as glucose. Glucose could indirectly induce the expression of *nupR* by promoting the function of CcpA. Thus, a glucose induction experiment was performed in SSM medium. The results showed that the transcription activity of *nupR* increased by 2.5-fold, which is consistent with the assumption. After the mutation of the conserved CcpA binding sites, it was not influenced by the addition of 0.1% glucose (Fig. 7D). These results further shed light on the regulation of NupR by CcpA.

For further verification, EMSA was performed. ComK, PurR, or CcpA protein with His tag was purified and mixed with the corresponding fragment of the *nupR* promoter containing ComK box (228-bp DNA fragment produced by PCR using primers ComKf-EMSA and ComKr-EMSA), PurR box (the DNA fragment was the same as the sequence containing the ComK box), or CcpA box (248-bp DNA fragment produced by PCR using primers CcpAf-EMSA and CcpAr-EMSA). Band shifts were all observed (Fig. 7E). Overall, ComK and PurR can directly and negatively regulate the expression of *nupR*. Conversely, CcpA directly and actively governs it.

### Regulation of *nupR* by a riboswitch.

**FIG 8:**
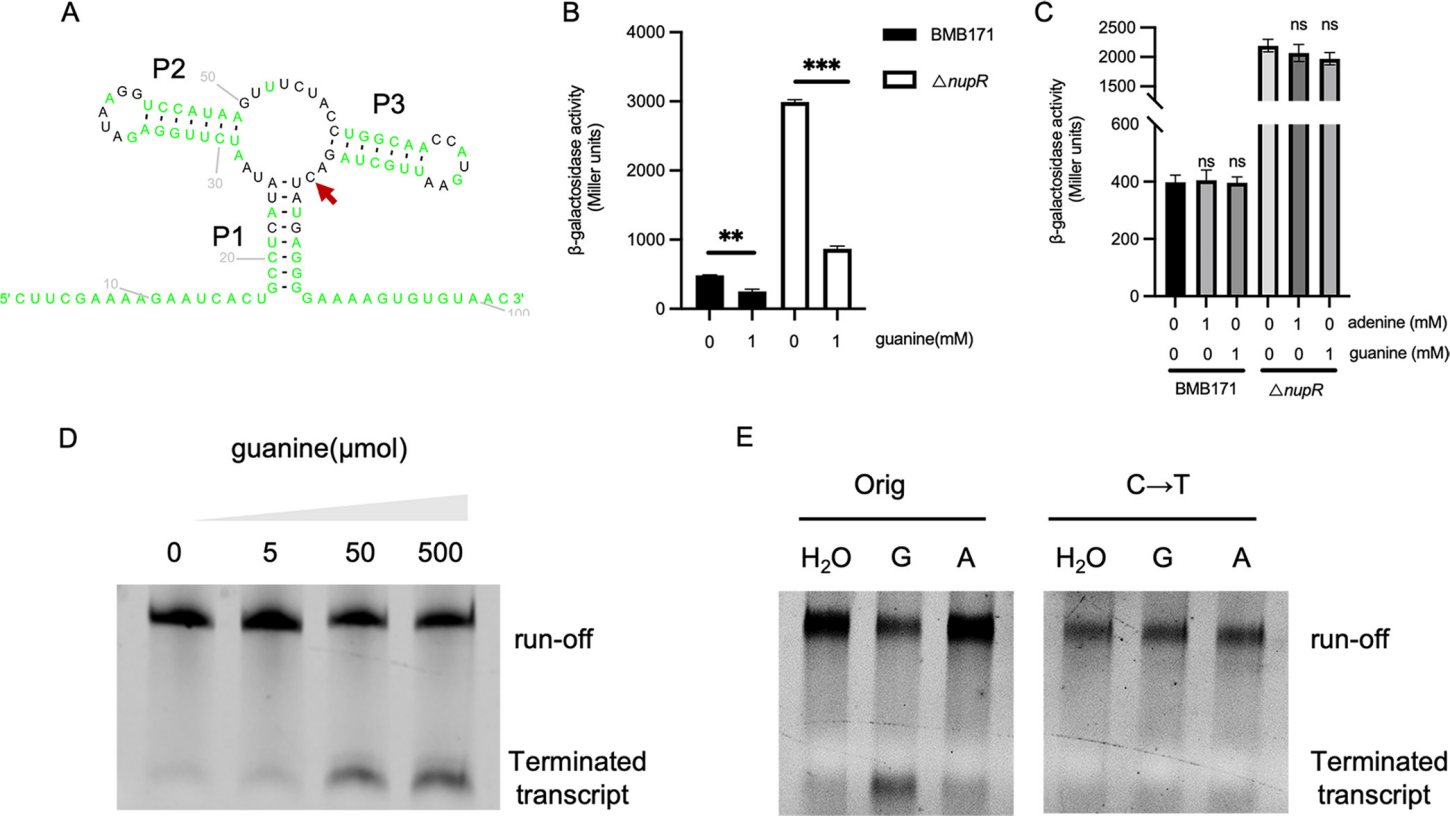
Regulation of riboswitch on NupR. (A) Structure of the purine riboswitch located in the 5′ UTR of *nupR* predicted by Rfam. The same sequence with the template is marked as black, and modified sequence compared to the template is marked as green. The red arrow marks the keg nucleobase cytosine that determines the purine riboswitch binding specificity. (B) Guanine represses the transcription activity of *nupR*-p14. β-Galactosidase activity of BMB171(pHT1K-*nupR*-p14) and △*nupR* (pHT1K-*nupR*-p14) in SSM medium with or without 1 mM guanine. **, *P < *0.01; ***, *P < *0.001 by Student's *t* test. Data represent the mean ± SD from three independent samples. (C) Transcription activity of *nupR*-p14 is unaffected by guanine or adenine after mutation of the key nucleobase C to T in BMB171 or △*nupR*. The β-galactosidase activity was assessed in the BMB171 strain and △*nupR* containing the pHT1K-*nupR*-p14CT in SSM medium in the presence of adenine/guanine or not. ns, not significant by Student's *t* test. Data represent the mean ± SD from three independent samples. (D) *In vitro* transcription with increasing guanine concentrations ranging from 0 to 500 mM using a DNA fragment from −582 to −35 (*nupR*-p14) as the template. (E) *In vitro* transcription was carried out by supplying 50 μmol guanine (marked as G) or adenine (marked as A) as a ligand and using DNA fragment *nupR*-p14 as the template (marked as Orig) or nupR-p14 with the critical nucleobase C mutant to T as the template (marked as C→T).

Besides transcription factor binding sites, a purine riboswitch is located upstream of *nupR*, and the structure is shown in Fig. 8A. Conserved nucleotides form the tight ligand-binding pocket of this aptamer class at the junction of three stems, termed P1, P2, and P3. The single nucleobase (guanine or adenine) between P2 and P3 determines the specificity of the riboswitch-binding ligand. After the single U→C mutation in the P1-P3 junction, the purine riboswitch binds guanine instead of adenine (19). Because the nucleobase between P2 and P3 of our predicted purine riboswitch was C, we speculated that the ligand of the purine riboswitch might be guanine.

pHT1K-*nupR*-p14CT with the single C→T mutation was constructed. Then, plasmids pHT1K-*nupR*-p14 and pHT1K-*nupR*-p14CT were introduced to the wild-type and the Δ*nupR* strains to generate BMB171(pHT1K-*nupR*-p14), BMB171(pHT1K-*nupR*-p14CT), △*nupR* (pHT1K-*nupR*-p14), and △*nupR* (pHT1K-*nupR*-p14CT) strains, respectively. Four strains were cultivated in SSM with or without guanine. When the strains reached the end of logarithmic growth, bacterial suspensions were collected for β-galactosidase activity. The results indicated that in the presence of guanine, the transcriptional activity of *nupR*-p14 reduced significantly in both the BMB171 and the Δ*nupR* (Fig. 8B). It suggested that the riboswitch ligand is guanine, and the riboswitch represses the expression of *nupR* by binding guanine. However, as the critical nucleobase of riboswitch C→T, neither guanine nor adenine could influence the transcriptional activity of *nupR*-p14 in BMB171 or Δ*nupR* (Fig. 8C).

To investigate the regulatory mechanisms of the riboswitch, we tested the possibility that the riboswitch could induce termination by adding exogenous guanine to the transcription reaction. *In vitro* transcription was carried out by supplying guanine as a ligand and using a DNA fragment from −582 to −35 (*nupR*-p14) as the template. Riboswitch-terminated transcripts were increased with growing concentrations of guanine, and the runoff transcripts were decreased accordingly (Fig. 8D). Riboswitch-terminated transcripts could not be observed with the addition of adenine, suggesting that guanine, but not adenine, is the riboswitch ligand. Also, when the critical nucleobase C of the DNA template was mutated to T, there was no riboswitch terminated transcript (Fig. 8E). Thus, guanine directly promotes intrinsic termination in the *nupR* promoter region. Overall, these results suggest that nucleoside C is indispensable for binding between guanine and riboswitch. Moreover, after binding guanine, the riboswitch represses the expression of *nupR*-p14 by enhancing the transcriptional termination.

## DISCUSSION

In this study, we demonstrated that the NupR mainly controls the expression of *nupC1*, *nupC2*, *nupC3*, *nupC4*, and the *nup* operon required for the utilization of nucleosides in B. thuringiensis BMB171. B. thuringiensis belongs to the Bacillus cereus groups, an essential clade of *Bacillus* species. In addition, Bacillus cereus also includes B. cereus and B. anthracis (20). The most significant difference among B. thuringiensis, B. cereus, and B. anthracis is that B. thuringiensis can produce insecticidal crystal proteins while producing spores. Moreover, B. thuringiensis is environmentally friendly and harmless to people and animals. In contrast, B. cereus and B. anthracis can produce pathogenic toxins in humans (21). After homology analysis, we discovered that the NupR protein is widely present in *Bacillus species*, especially in Bacillus cereus groups. It also exists in Clostridioides difficile, Pseudomonas aeruginosa, Streptococcus pneumoniae, etc. (Fig. 1). Thus, this study could provide essential clues for future studies exploring the function of the NupR-like proteins in those strains.

Moreover, BLAST analysis dedicated that the riboswitch in the 5′ noncoding region of *nupR* was limited to the 5′ noncoding region of the *gntR* locus within the chromosome of Bacillus cereus groups (see Table S2 in the supplemental material). The mechanism that regulates *gntR* by this riboswitch may be widespread in Bacillus cereus groups. It could have essential reference significance for the Bacillus anthracis and Bacillus cereus pathogenic bacteria.

Riboswitches are a new class of genetic regulatory elements found in the 5′ UTR in bacterial messenger RNAs, and they consist of two domains, namely, the expression platform and the aptamer domain (22, 23). After the aptamer responds to ligands such as ions, purines, amino acids, and enzyme cofactors, the structure of the expression platform can transform to modulate transcription antitermination or ribosome binding to regulate the expression of a downstream gene without the involvement of any protein (24, 25). The downstream genes regulated by the riboswitch are usually directly related to the formation of corresponding physiological and biochemical signals. Most riboswitches regulate the synthesis of various primary metabolites and the expression of multiple transporters, but relatively few control transcription factors (26). Existing studies have reported that the toxicity regulator PrfA in Listeria monocytogenes is regulated by temperature-sensitive ribose switches in its upstream noncoding region (27). Two *S*-adenosylmethionine (SAM) riboswitches can also bind to the upstream noncoding region of *prfA* and regulate the expression (28). In addition, the vitamin B_12_ riboswitch regulates the expression of PocR transcription factors by transcribing a portion of the antisense RNA, thereby controlling pathogenic infections (29). In this study, the riboswitch regulates the transcription of the downstream gene encoding a transcription regulation factor, NupR, by enhancing the transcriptional termination.

Ligand-binding aptamer domains and expression platforms form the typical riboswitch structure. The aptamer domain adopts a complex structure fold to form a conserved receptor for the ligand. Computer-aided searching has classified representative riboswitches based on conserved RNA sequences and structure. We predicted that the riboswitch in this research was a purine riboswitch by using the riboswitch prediction software Rfam (http://rfam.xfam.org/). Various studies have indicated that a single mutation (C to U) within the guanine riboswitches changes ligand binding specificity to adenine rather than guanine (30–33). However, after changing the core site of the riboswitch (C to U), adenine did not affect the expression of *nupR*, and the riboswitch-terminated transcript was not observed with adding adenine (Fig. 8C and E). Hence, it is slightly different from the previously reported riboswitches. The properties of this purine riboswitch need to be further investigated.

We found that the riboswitch can downregulate the expression of *nupR* by binding to the ligand guanine and promoting the transport of extracellular nucleosides into the cytosol. In Fig. 8D, the terminated transcript was observed when the guanine concentration reached 50 μmol but was not noticeable when the guanine concentration was 5 μmol. Previous studies have shown that it did not affect the growth of Jurkat E6-1 cells when the intracellular guanine concentration was below 10 μmol. Nevertheless, as the concentration of guanine was above 10 μmol, it affected the intracellular metabolism of adenosine, cytidine, and thymidine and inhibited DNA synthesis and cell growth. However, adding other nucleosides, especially adenosine, can effectively eliminate the toxicity of high guanine concentrations by relieving the DNA synthesis inhibition and restoring it to normal (34, 35). Therefore, when the intracellular concentration of guanine reaches a certain threshold, the riboswitch could bind to it, inhibit the transcriptional activity of *nupR*, and promote the expression of nucleoside permeases, which can transport other classes of nucleosides into the cells and reduce the toxicity of guanine to the cells. In this process of regulating, NupR and its upstream riboswitch may play a tightly regulatory role and govern the balance of intracellular nucleoside concentration and homeostasis.

Guanine deaminase, a Zn metalloenzyme, can irreversibly deaminate guanine to produce xanthine. It can reduce intracellular guanine concentration and decrease the toxicity of high guanine concentrations (36, 37). When the zinc ions concentration rises, the intracellular guanine decreases accordingly. It dissociates NupR from the target DNA as the concentration of zinc ions reaches a certain threshold. Thus, the expression of nucleoside transport-related protein could increase, leading to the uptake of guanosine, adenosine, cytidine, and uridine and maintaining the intracellular nucleoside homeostasis.

In addition to self-regulation, the expression of *nupR* is regulated by the transcription factors CcpA, ComK, and PurR. CcpA (catabolite control protein), which belongs to the LacI family, is a central regulator of carbon metabolism in B. subtilis. CcpA can mediate the cellular carbon catabolite repression (CCR) effect (38). In the presence of preferred carbon sources such as glucose, HPrK/P (Hprkinase/phosphoesterase) kinase, which phosphorylates the serine residues of HPr (histidine-phosphoryl protein) to P-(Ser)-HPr, can be activated by high intracellular concentrations of ATP, fructose-1,6-bisphosphate (FBP), and glucose-6-phosphate (G-6-P). With the assistance of P-(Ser)-HPr, CcpA can bind to the catabolite responsive element (*cre*) site of the target genes and block the utilization of less favored sources of carbon (18). As shown in Fig. 7C and D, the transcriptional activity of *nupR* decreased significantly with the *cre* site mutation and increased with 0.1% glucose. Hence, we speculated that, as glucose was present, CcpA promoted the expression of *nupR* and inhibited the expression of nucleoside permeases, preferentially utilizing glucose rather than nucleoside analogs.

PurR also belongs to the LacI family, which negatively regulates the expression of NupR. The classical role of PurR is to negatively control the production of purine. Phosphoribosylpyrophosphate (PRPP), which is involved in synthesizing nucleotides, can inhibit the binding between PurR and the *pur* operator site and promote the generation of nucleotides (12). Meanwhile, by negatively regulating the expression of *nupR*, PurR could accelerate the utilization of extracellular nucleotides instead of self-synthesis.

Moreover, competence transcription factor (ComK) is the critical regulator in competence development in B. subtilis. At the beginning of stationary growth in place of exponential growth, ComK levels increase and upregulate the transcriptional activity of genes involved in DNA uptake (39, 40). When the ComK binding site was mutated, the expression of *nupR* significantly increased (Fig. 7C). Hence, we infer that, in competence development, ComK could inhibit the expression of *nupR* and then promote nucleoside transport into cells. The results suggested that the expression of *nup*R responds to multiple signals, the intracellular nucleoside pool, the PRPP pool responded by PurR, preferred carbon sources monitored by CcpA, and cell competence development regulated mainly by ComK. Then, NupR could regulate nucleoside transport to maintain the intracellular nucleoside concentration balance.

Nucleosides are essentially glycosides, which are components of nucleic acids and nucleotides. Nucleoside utilization and homeostasis are vital for cell growth and proliferation. Also, in recent years, it has been found that nucleosides/peptides could improve skin appearance and inhibit the aging process by signaling fibroblasts to stimulate collagen production in the dermis. Therefore, nucleosides are used in pharmaceuticals (41). Moreover, nucleosides modulate the catalytic activity of metal nanoparticles. For example, nucleosides improve the catalytic activity of copper nanoparticles, which have been applied to detect mercury ions (42). More critically, nucleosides and nucleotide analogs have been used as the most prominent antiviral drugs for the treatment of herpes simplex virus (HSV), human immunodeficiency virus type 1 (HIV-1), and human hepatitis B (HBV). More than 20 FDA-approved nucleoside and nucleotide analogs are used as antiviral agents for multiple infections (43, 44). Therefore, researchers have been devoted to constructing nucleoside/nucleoside analog-producing engineered strains. Understanding the genetic mechanism of intracellular nucleoside metabolism and transport will promote such research. For example, Bacillus subtilis is a commercial strain that produces cytidine and uridine. After genetic modification of nucleoside metabolism and transport genes, Hui Zhu et al. increased the production of cytidine and uridine by 259.5% and 11.2%, respectively (45).

Nucleosides are not only required for bacterial growth and proliferation but also have broad application prospects in industry and medicine. In order to increase the yield of nucleosides, it is essential to study the mechanisms of nucleoside metabolism and transport regulation. Transcription factors CRP, DeoR, CytR, and PurR have been verified to control nucleoside metabolism and transport (46). NupR, in this study, is the first transcription factor of the GntR family identified to be involved in the regulation of nucleoside transport. This study will provide new theoretical guidance for constructing nucleoside-producing engineering strains.

## MATERIALS AND METHODS

### Bacterial strains and culture conditions.

The bacterial strains and plasmids used in this study are listed in Table S3 in the supplemental material. Escherichia coli DH5α strains were cultured at 37°C in Luria-Bertani (LB) medium in an orbital shaker (200 rpm). Bacillus thuringiensis strain BMB171 and its knockout strains were generally cultured at 28°C with shaking at 200 rpm in LB medium or Schaeffer’s sporulation medium (SSM) (47). Medium with a sole carbon source was prepared in M9 medium (6.78 g/mL Na_2_HPO_4_, 3 g/mL KH_2_PO_4_, 0.5 g/mL NaCl, 1 g/mL NH_4_Cl, 0.241 g/mL MgSO_4_, and 0.011 g/mL CaCl_2_) with 1 mM guanosine or other nucleosides. The final antibiotic concentrations used for bacterial selection were 100 mg/mL ampicillin, 5 mg/mL erythromycin, and 100 mg/mL kanamycin.

### Construction of the Δ*nagR2* and complemented strains.

The Δ*nagR2* was constructed using the gene knockout method (48). The upstream and downstream DNA fragments (named *nagR2*L and *nagR2*R, respectively) of *nagR2* were amplified using PCR with the primers *nagR2*L-F, *nagR2*L-R, *nagR2*R-F, and *nagR2*R-R, which are listed in Table S3. *nagR2*L and *nagR2*R were cloned into the temperature-sensitive plasmid pRP1028 via SmaI-MluI and MluI-BamHI sites, respectively, and produced plasmid pRP1028-*nagR2*LR. Then, the plasmid was transferred into the BMB171 strains by electroporation for knockout construction. Subsequent procedures were performed as previously described, and the strain BMB171Δ*nagR2* was finally obtained (49). To verify that *nagR2* was knocked out, genomic DNA was extracted from the strain Δ*nagR2* and served as the template for PCR by using the primers *nagR2*1 and *nagR2*2 listed in Table S3, and then the RCR product was sequenced. The sequencing results were correct, and there was no base mutation, indicating that *nagR2* was knocked out.

In order to construct the complemented Δ*nagR2* strain, the *nupR* (*nagR2*) fragment was amplified from the BMB171 genome using primers *nupR*com-F and *nupR*com-R. The fragment was digested with EcoRI and XbaI and inserted into the corresponding sites of pKSV7 to create the pKSV7-P*nupR*-*nupR*-complementing plasmid. The plasmid was transferred into BMB171 by electroporation (50). The complementary strains were screened by PCR detection and sequencing verification.

### RNA extraction, cDNA synthesis, and quantitative real-time reverse transcription-PCR analysis.

Total RNA of BMB171 and Δ*nagR2* strain was extracted using TRIzol reagent (Invitrogen, Waltham, MA, USA). cDNA was synthesized from 1 μg of total RNA using a PrimeScript reverse transcription (RT) reagent kit with gDNA Eraser (TaKaRa, Shiga, Japan) according to the instructions. Then, the synthesized cDNA was used as the template for quantitative real-time PCR (qPCR) using TB green premix Ex Taq II (TaKaRa) and StepOnePlus real-time PCR system (Applied Biosystems, Foster City, CA, USA) according to the manufacturer’s instructions. The 16S rRNA gene was used as an internal control to normalize the relative expression level of each gene and finally calculated using the threshold cycle (2^−△△^*^CT^*) method (51).

### Transcriptome assay and data analysis.

BMB171 and BMB171△*nagR2 cells* were cultured in LB medium at 28°C and 200 rpm, harvested at the exponential phase, frozen in liquid nitrogen immediately, and stored at −80°C until use. The samples (BMB171 and BMB171△*nagR2*) were shipped to Genewiz (www.genewiz.com) for mRNA-seq library construction and transcriptome sequencing. As previously described, mRNA-seq library construction, Solexa sequencing, and RNA-seq data analysis were performed (4).

### EMSA.

The expression and purification of NagR2, ComK, CcpA, and PurR were performed according to previously described methods (4).

The electrophoretic mobility shift assay was performed as previously described with minor modifications (52). DNA fragments were incubated with purified recombinant protein NagR2 at 25°C for 30 min in a 10-μL reaction mixture [10 mM Tris-HCl, pH 7.5, 50 mM NaCl, 0.5 mM dithiothreitol (DTT), 4% glycerol, 200 ng poly(dI-dC)]. However, regarding the ComK, CcpA, and PurR proteins, DNA fragments were incubated in a 10-μL reaction mixture [10 mM Tris-HCl, pH 8.0, 50 mM KCl, 10 mM EDTA, 4% glycerol, and 500 ng poly(dI-dC)]. The mixture was loaded onto a 6% native polyacrylamide gel in 0.5× TBE buffer (0.044 M Tris base, 0.044 M boric acid, and 0.001 M EDTA, pH 8.0), which had been prerun for 30 min on ice at 100 V and run at 140 V for 90 min. Band bands were visualized with a ChemiDoc XRS+ (Bio-Rad) molecular imager for DNA labeled with biotin.

EMSA was further performed using Zn^2+^ (0, 0.04, 0.2, 1, and 5 mM) or Cu^2+^ (0, 0.5, 1, and 5 mM) at different concentrations, 50 ng DNA, and 8 ng NupR to research the ligand of NupR. Moreover, the following experiments were utilized to exclude the possibility of denaturation of proteins by metal ions. We added water, binding buffer, NupR protein, metal ions, EDTA, and DNA to the reaction in order. When adding the last four samples, the reaction was left for 15 min each time the sample was added. For the negative control, we added water instead of EDTA.

### Plasmid construction.

β-Galactosidase activity analysis-related plasmids were constructed based on pHT1K containing the reporter *lacZ*. The target DNA fragment *nupR*-p14 was generated by PCR using primers *nupR*-p14F/R containing homologous arms of pHT1K listed in Table S3. The DNA fragment *nupR*-p14CTu was generated by PCR using primers *nupR*-p14CTuF and *nupR*-p14CTuR containing a mutant (“C”→“T”) and *nupR*-p14CTd using primers *nupR*-p14CTdF containing a mutant (C→T) and *nupR*-p14CTdR (Table S3). The *nagR2*-p14CT fragment was generated by overlapping PCR using the primers *nupR*-p14CTuF and *nupR*-p14CTdR containing homologous arms of pHT1K with the template *nupR*-p14CTu and *nupR*-p14CTd. PCR products containing the promoters *nupR*-p14 and *nupR*-p14CT were ligated into pHT1K via the SalI-NcoI sites to generate plasmids pHT1K-*nupR*-p14 and pHT1K-*nupR*-p14CT (Table S3). Also, pHT1K-*nupR*-p14-CcpAmu, pHT1K-*nupR*-p14-PurRmu, and pHT1K-*nupR*-p14-ComKmu were also constructed, respectively, using primers ccpAf1/r1/f2/r2, purRf1/r1/f2/r2/f3/r3, and comKf1/f2/r1/r2.

NagR2, PurR, and ComK with His tags were purified from E. coli BL21(DE3). The plasmids pET*nagR2*, pET*purR*, and pET*comK* were constructed with PCR amplification of *nagR2*, *purR*, and *comK* from the BMB171 genome using the primer pairs *nagR2*-F/R, *purR*-F/R, and *comK*-F/R (Table S3), all containing the homologous arms of pET28a(+). By seamless cloning, the DNA fragment was cloned into plasmid pET28a(+) and then transferred into E. coli BL21(DE3) to produce BL21 (pET*nagR2*), BL21 (pET*purR*), and BL21 (pET*comK*). BL21-pET-CcpA was constructed in a previous study and used to purify the CcpA protein with a His tag (5).

### β-Galactosidase activity analysis.

B. thuringiensis cells were grown in SSM medium with shaking (200 rpm at 28°C). For the riboswitch ligand research, guanine or adenine was added at a final concentration of 1 mM. To investigate the ligand of NupR, Zn^2+^ was supplemented at the final concentrations of 0, 50 nM, 500 nM, 5 μM, or 50 μM. Then, 4 mL cells at the exponential growth phase (OD_600_ = 0.55) were collected and centrifuged (12,000 × *g* for 1 min). The pellets were used to measure the β-galactosidase activity using approaches described elsewhere (53).

### *In vitro* transcription.

Normal DNA of the *nupR* promoter was generated with PCR amplification from the BMB171 genome using nupR-p14F/R primers, but C→T mutant DNA was amplified from the DNA fragment *nupR*-p14CT. *In vitro* transcription was performed as previously described (54). Increasing guanine was added to the reaction mixture to obtain final concentrations of 5 μm, 50 μm, or 500 μm. Water or adenine was added as a negative control.

### 5′ Rapid amplification of cDNA ends.

In order to identify the transcription start site (TSS) of the *nupR* gene, a 5′-RACE experiment was performed as described previously (55).

### Nucleoside utilization.

Twenty milliliters of M9 medium without glucose containing different nucleosides at a final concentration of 1 mM were inoculated with 1% overnight culture of B. thuringiensis strains and grown at 28°C and 200 rpm. Note that the initial OD_600_ was 0.01. One milliliter of culture was taken at an interval of 2 h to determine the OD_600._ However, due to the poor medium, BMB171 cells converged over 4 h, and we could only obtain the OD_600_ value at the 4th hour. The data shown are the mean values of at least three independent experiments.

In addition, we determined the sensitivity of B. thuringiensis strains to toxic 5-fluorouridine. One hundred milliliters of LB medium were inoculated with 1 mL overnight culture of B. thuringiensis strains and grown at 28°C and 200 rpm. In the middle of the logarithm, 1 mL bacterial solution was taken, diluted, and dropped into LB solid medium with or without 5-fluorouridine nucleoside at a final concentration of 20 μg/mL.

### Data availability.

The RNA-seq data are available in the BioProject database with accession number PRJNA825124.
