# Supplementary material for: NupR Responding to Multiple Signals Is a Nucleoside Permease Regulator in Bacillus thuringiensis BMB171
Source: Microbiol Spectr. 2022 Jul 7;10(4):e01543-22. doi: 10.1128/spectrum.01543-22 (PMC9430930; doi:10.1128/spectrum.01543-22)
Supplement: Supplemental file 1 — Fig. S1-S3; Tables S1-S3. Download spectrum.01543-22-s0001.pdf, PDF file, 0.5 MB [file spectrum.01543-22-s0001.pdf]

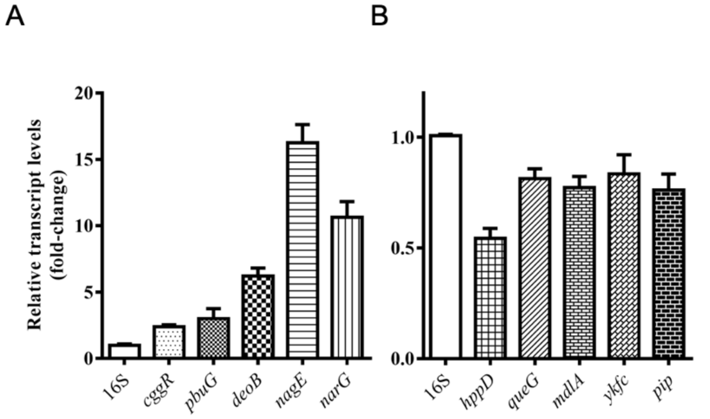

Figure S1. Validation of RNA-seq data by qRT-PCR. (A). Five randomly selected DEGs with elevated expression, including *cggR* (BMB171_RS25590), *pbuG* (BMB171_RS20290), *deoB* (BMB171_RS20310), *nagE* (BMB171_RS02520), *narG* (BMB171_RS10500). (B). Five randomly selected DEGs with reduced expression, including *hppD* (BMB171_RS01295), *queG* (BMB171_RS02835), *mdlA* (BMB171_RS04395), *ykfc* (BMB171_RS14010), *pip* (BMB171_RS23225). The values in the graphs are the mean of three independent replications, and the error lines indicate the standard deviation.

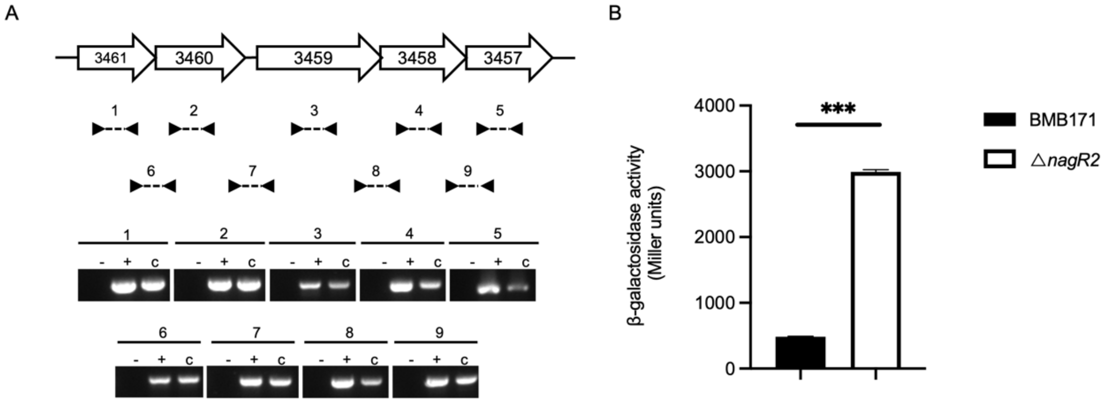

Figure S2. (A). Identification of the co-transcribed of *nup.* 1-5, Internal fragments of the CDSs of *nup* operon. 6-9, Fragments between adjacent genes of *nup* operon. -, negative control, water was added as temple. +, positive control, genome DNA was added as temple. c, experimental group, cDNA was added as temple. 3461-3457: BMB171_C3461 (NagR2), BMB171_C3460 (nucleoside-binding protein), BMB171_C3459 (nucleoside transport ATP-binding protein), BMB171_C3458-3457 (nucleoside transport system permease protein). (B). NagR2 inhibits the expression of *nup* operon. The β-galactosidase activity of BMB171(pHT1K-*nagR2*-p14) and △*nagR2* (pHT1K-*nagR2*-p14) in SSM medium. ****P* < 0.001 by Student's *t* test. Data represent the mean ± SD from three independent samples.

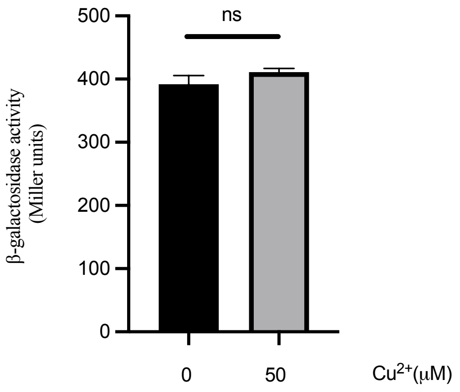

Figure S3. The transcriptional activity of *nupR* is not affected by the copper ions. The β-galactosidase activity of BMB171(pHT1K-nagR2-p14) in SSM medium with or without Cu^{2+}.ns, not significant by Student's *t* test. Data represent the mean ± SD from three independent replicates.

Table S1 Genes controlled by NagR2 in Bt BMB171.

locus_tag	desprition	fold-change	*nup site*
**Genes up-regulated in the *ΔnagR*2 mutant compared to wild-type strain BMB171**			
BMB171_RS01930	nucleoside permease nupC, *nupC*1	5.03	√[a]
BMB171_RS02520	PTS system, N-acetylglucosamine-specific IIBC component, *nagE*	1.74	×[b]
BMB171_RS03885	potassium-transporting ATPase subunit B	1.60	[c]
BMB171_RS06420	Sodium export ATP-binding protein	2.05	×
BMB171_RS09285	amino acid permease	2.44	×
BMB171_RS09485	L-lactate dehydrogenase, *ldh*	2.03	√
BMB171_RS10120	branched chain amino acid ABC transporter carrier protein, *brnQ*	1.80	×
BMB171_RS10500	respiratory nitrate reductase alpha chain, *narG*	2.80	×
BMB171_RS10505	respiratory nitrate reductase beta chain, *narH*	2.81	×
BMB171_RS10510	respiratory nitrate reductase delta chain, *narJ*	2.78	×
BMB171_RS10515	respiratory nitrate reductase delta chain, *narI*	2.68	×
BMB171_RS10525	molybdenum cofactor biosynthesis protein A, *moaA*	2.27	×
BMB171_RS10530	thiamine/molybdopterin biosynthesis MoeB-like protein, *moeB*	1.91	×
BMB171_RS10535	molybdopterin biosynthesis protein MoeA, *moeA*	2.19	×
BMB171_RS10540	molybdopterin (MPT) converting factor, subunit 2, *moaE*	1.88	×
BMB171_RS10550	nitrite extrusion protein	2.32	×
BMB171_RS10575	CbiX protein	1.51	
BMB171_RS10580	bifunctional uroporphyrinogen-III methyltransferase	1.65	×
BMB171_RS10585	nitrite reductase [NAD(P)H] small subunit, *nirD*	1.72	×
BMB171_RS10590	nitrite reductase [NAD(P)H] large subunit, *nirB*	1.89	×
BMB171_RS11670	cold shock protein	2.25	×
BMB171_RS16385	Extracellular ribonuclease	2.36	×
BMB171_RS18055	Sodium/proline symporter	6.56	×
BMB171_RS20290	guanine-hypoxanthine permease, *pbuG*	1.45	×
BMB171_RS20300	pyrimidine-nucleoside phosphorylase, *pyn*	2.54	×
BMB171_RS20305	purine nucleoside phosphorylase, *punA*	2.56	×
BMB171_RS20310	phosphopentomutase, *deoB*	2.72	×
BMB171_RS20570	hypothetical protein	1.68	
BMB171_RS25245	nucleoside transporter NupC family, *nupC*2	5.36	√
BMB171_RS25250	nucleoside transporter NupC family, *nupC*3	5.03	√
BMB171_RS25585	glyceraldehyde-3-phosphate dehydrogenase, *gap*2	1.80	×
BMB171_RS25590	central glycolytic genes regulator, *cggR*	2.63	×

BMB171_RS25960	6-phospho-beta-glucosidase, *celf*	2.07	✕
BMB171_RS25970	PTS system lichenan oligosaccharide-specific transporter subunit IIC, celB2	1.77	✕
BMB171_RS26110	nucleoside transporter NupC family, *nupC*4	5.40	√
BMB171_RS26135	enterotoxin/cell-wall binding protein	2.63	✕
BMB171_RS26735	bacillolysin, *baci*	1.45	√
BMB171_RS26950	HAD superfamily hydrolase	1.71	√
**Genes down-regulated in the Δ*nupR* strain compared to wild-type strain BMB171**			
BMB171_RS01295	4-hydroxyphenylpyruvate dioxygenase, *hppD*	-1.963	✕
BMB171_RS01300	fumarylacetoacetase, *fahA*	-2.023	✕
BMB171_RS01305	homogentisate 1,2-dioxygenase, *hmgA*	-1.521	✕
BMB171_RS01310	amino acid permease	-1.513	✕
BMB171_RS01970	Na+/H+ antiporter, *nhaC*	-1.658	✕
BMB171_RS02835	(Fe-S)-binding protein, *queG*	-2.472	✕
BMB171_RS02840	hypothetical protein	-2.289	
BMB171_RS03130	vancomycin B-type resistance protein vanW	-1.528	✕
BMB171_RS03475	hypothetical protein	-1.538	
BMB171_RS03535	proline-specific permease	-1.711	✕
BMB171_RS04155	oligopeptide transport system permease OppB	-2.332	✕
BMB171_RS04160	oligopeptide transport system permease OppC	-2.816	✕
BMB171_RS04240	amino acid permease	-1.892	✕
BMB171_RS04395	multidrug ABC transporter permease, *mdlA*	-2.087	✕
BMB171_RS05350	hypothetical protein	-2.318	
BMB171_RS05540	hypothetical protein	-1.818	
BMB171_RS05560	long chain fatty acid CoA ligase	-1.997	✕
BMB171_RS05735	malate synthase, *aceB*	-1.545	✕
BMB171_RS05740	isocitrate lyase, *aceA*	-2.161	✕
BMB171_RS05875	Ammonia channel	-1.747	
BMB171_RS06265	hypothetical protein	-2.361	
BMB171_RS06655	PHA synthase PhaC	-1.469	
BMB171_RS07100	ATP phosphoribosyltransferase regulatory subunit	-3.166	✕
BMB171_RS07685	Hypothetical cytosolic protein	-2.063	
BMB171_RS08170	Na+/H+ antiporter NapA	-1.475	✕
BMB171_RS08855	response regulator aspartate phosphatase	-1.611	✕
BMB171_RS10015	oligopeptide-binding protein OppA	-2.674	
BMB171_RS10110	hypothetical protein	-2.091	
BMB171_RS10490	hypothetical protein	-1.582	
BMB171_RS11015	oligopeptide transport system permease OppC	-1.965	
BMB171_RS11165	lysine 2,3-aminomutase, *kamA*	-2.587	✕
BMB171_RS11665	cold shock protein	-1.773	✕
BMB171_RS12330	short chain acyl-CoA dehydrogenase	-1.596	✕
BMB171_RS12655	ABC transporter ATP-binding protein	-1.536	✕
BMB171_RS12665	ABC transporter ATP-binding protein	-1.771	✕
BMB171_RS12670	ABC transporter permease	-1.55	✕

BMB171_RS13125	macrolide-efflux protein	-2.033	×
BMB171_RS13815	hypothetical protein	-2.318	
BMB171_RS13820	PadR family transcriptional regulator	-Inf	×
BMB171_RS14005	oligopeptide-binding protein OppA	-3.665	×
BMB171_RS14010	cell wall-associated hydrolase, *ykfc*	-3.615	×
BMB171_RS14015	muconate lactonizing family protein	-3.326	
BMB171_RS14720	immune inhibitor A precursor	-2.009	×
BMB171_RS15245	kinase autophosphorylation inhibitor kipI	-2.045	×
BMB171_RS15320	Phage infection protein	-2.429	
BMB171_RS15940	macrolide-efflux protein	-1.478	
BMB171_RS16320	hypothetical protein	-2.166	
BMB171_RS16325	NAD(P)H dehydrogenase, quinone family	-2.371	
BMB171_RS16330	SWIM zinc finger containing protein	-3.002	×
BMB171_RS17760	oligopeptide-binding protein OppA	-2.343	
BMB171_RS17765	oligopeptide-binding protein OppA	-2.477	
BMB171_RS17775	proton/sodium-glutamate symport protein	-1.567	
BMB171_RS17985	medium chain fatty acid CoA ligase, *alkK*	-2.066	×
BMB171_RS18065	N-ethylmaleimide reductase	-2.014	
BMB171_RS18070	transcriptional regulator, *carD*	-2.118	×
BMB171_RS18360	glutamine synthetase, type I, *glnA*	-1.718	×
BMB171_RS18365	MerR family transcriptional regulator, *glnR*	-1.726	×
BMB171_RS18455	GABA permease, *gabP*	-2.679	×
BMB171_RS18795	GntR family transcriptional regulator, *nagR2*	-3.95	√
BMB171_RS19090	ACP S-malonyltransferase	-1.674	×
BMB171_RS19545	cytochrome c oxidase, subunit IVB	-3.68	
BMB171_RS19645	hypothetical protein	-2.707	
BMB171_RS19885	sporulation kinase B, *kinB*	-1.496	×
BMB171_RS19960	hypothetical protein	-1.62	
BMB171_RS20040	2,3-diketo-5-methylthiopentyl-1-phosphate enolase, *mtnW*	-1.727	×
BMB171_RS20060	hypothetical protein	-1.993	
BMB171_RS20230	Sodium/proline symporter	-3.19	×
BMB171_RS20640	arginine ABC transporter permease, *artQ*	-2.854	
BMB171_RS21225	bifunctional homocysteine S-methyltransferase, *mmuM*	-2.453	×
BMB171_RS21430	transcriptional repressor CcpN	-1.45	×
BMB171_RS22795	S-adenosylmethionine decarboxylase	-1.435	
BMB171_RS22800	glyceraldehyde-3-phosphate dehydrogenase	-1.676	
BMB171_RS22965	hypothetical protein	-2.439	
BMB171_RS23225	proline iminopeptidase, *pip*	-1.884	×
BMB171_RS25255	Sodium/proton-dependent alanine carrier protein	-1.58	
BMB171_RS26465	NADH dehydrogenase subunit B, *nuoB*	-2.073	
BMB171_RS26695	3-hydroxybutyryl-CoA dehydrogenase	-1.79	
BMB171_RS26700	Acetyl-CoA acetyltransferase	-2.111	

BMB171_RS27215	hemolysin III	-1.771	×
BMB171_RS28250	acetolactate synthase large subunit	-2.044	×
BMB171_RS28255	AMP-dependent synthetase and ligase, *fadD*	-2.11	×
BMB171_RS28265	ketoacyl-ACP synthase, *fabF*	-2.124	×
BMB171_RS28270	cyclic peptide transporter, *pvdE*	-2.58	×
BMB171_RS28275	hypothetical protein	-1.968	
BMB171_RS29850	hypothetical protein	-2.259	

[a] Existent NupR binding site;[b] Not existent NupR binding site;[c] Not verified by EMSA

Table S2 Results of nucleotide blast analysis of nucleic acid sequences of the riboswitch

Description	Scientific Name	Query Cover	E value	Per. ident	Acc. Len	Accession
Bacillus mycoides strain DSM 2048 chromosome, complete genome	Bacillus mycoides	100%	4.00E-44	100	5285600	CP093291.1
Bacillus mycoides strain LX30 chromosome, complete genome	Bacillus mycoides	100%	4.00E-44	100	5462895	CP092833.1
Bacillus cereus strain IIPK35 chromosome	Bacillus cereus	100%	4.00E-44	100	5269886	CP068984.1
Bacillus anthracis strain Vollum chromosome, complete genome	Bacillus anthracis	100%	4.00E-44	100	5229859	CP076225.1
Bacillus anthracis strain A159 chromosome, complete genome	Bacillus anthracis	100%	4.00E-44	100	5228443	CP076222.1
Bacillus anthracis strain AF039 chromosome, complete genome	Bacillus anthracis	100%	4.00E-44	100	5209654	CP076219.1
Bacillus anthracis strain SA020 chromosome, complete genome	Bacillus anthracis	100%	4.00E-44	100	5220564	CP076216.1
Bacillus anthracis strain A0777 chromosome, complete genome	Bacillus anthracis	100%	4.00E-44	100	5207027	CP076213.1
Bacillus anthracis strain A1074 chromosome, complete genome	Bacillus anthracis	100%	4.00E-44	100	5227949	CP076210.1
Bacillus anthracis strain Pollino 3734 chromosome, complete genome	Bacillus anthracis	100%	4.00E-44	100	5228919	CP076207.1
Bacillus anthracis strain 3-IZSLT chromosome, complete genome	Bacillus anthracis	100%	4.00E-44	100	5229081	CP076204.1
Bacillus anthracis strain A3783 chromosome, complete genome	Bacillus anthracis	100%	4.00E-44	100	5279066	CP076201.1
Bacillus anthracis strain Tangail-4/2 chromosome, complete genome	Bacillus anthracis	100%	4.00E-44	100	5228141	CP076198.1
Bacillus anthracis strain 188678-1 chromosome, complete genome	Bacillus anthracis	100%	4.00E-44	100	5228516	CP076195.1
Bacillus anthracis strain UR-1 chromosome, complete genome	Bacillus anthracis	100%	4.00E-44	100	5229839	CP076192.1
Bacillus anthracis strain Pasteur chromosome, complete genome	Bacillus anthracis	100%	4.00E-44	100	5197747	CP076190.1
Bacillus anthracis str. BF1 chromosome, complete genome	Bacillus anthracis str. BF1	100%	4.00E-44	100	5213532	CP076187.1
Bacillus anthracis strain A49 chromosome, complete genome	Bacillus anthracis	100%	4.00E-44	100	5203814	CP076184.1
Bacillus anthracis strain A27 chromosome, complete genome	Bacillus anthracis	100%	4.00E-44	100	5230210	CP076181.1
Bacillus anthracis strain BUL 12 chromosome, complete genome	Bacillus anthracis	100%	4.00E-44	100	5230030	CP076178.1

Bacillus anthracis strain BUL 32 chromosome, complete genome	Bacillus anthracis	100%	4.00E-44	100	5229748	CP076176.1
Bacillus anthracis strain A178 chromosome, complete genome	Bacillus anthracis	100%	4.00E-44	100	5224614	CP076173.1
Bacillus anthracis strain A168 chromosome, complete genome	Bacillus anthracis	100%	4.00E-44	100	5230079	CP076170.1
Bacillus anthracis strain A166 chromosome, complete genome	Bacillus anthracis	100%	4.00E-44	100	5229216	CP076167.1
Bacillus anthracis strain A193a chromosome, complete genome	Bacillus anthracis	100%	4.00E-44	100	5229583	CP076164.1
Bacillus anthracis strain BUL 14 chromosome, complete genome	Bacillus anthracis	100%	4.00E-44	100	5229919	CP076163.1
Bacillus anthracis strain 4-IZSLT chromosome, complete genome	Bacillus anthracis	100%	4.00E-44	100	5229980	CP076160.1
Bacillus anthracis strain A87 chromosome, complete genome	Bacillus anthracis	100%	4.00E-44	100	5229688	CP076157.1
Bacillus anthracis strain BUL 16 chromosome, complete genome	Bacillus anthracis	100%	4.00E-44	100	5230027	CP076154.1
Bacillus anthracis strain BUL 31 chromosome, complete genome	Bacillus anthracis	100%	4.00E-44	100	5229640	CP076151.1
Bacillus anthracis strain BUL 19 chromosome, complete genome	Bacillus anthracis	100%	4.00E-44	100	5228566	CP076148.1
Bacillus anthracis strain BUL 40 chromosome, complete genome	Bacillus anthracis	100%	4.00E-44	100	5230038	CP076146.1
Bacillus anthracis strain 3016 chromosome, complete genome	Bacillus anthracis	100%	4.00E-44	100	5228841	CP076144.1
Bacillus anthracis strain A29 chromosome, complete genome	Bacillus anthracis	100%	4.00E-44	100	5229955	CP076141.1
Bacillus anthracis strain A182 chromosome, complete genome	Bacillus anthracis	100%	4.00E-44	100	5229323	CP076138.1
Bacillus anthracis strain Tyrol 3520 chromosome, complete genome	Bacillus anthracis	100%	4.00E-44	100	5210044	CP076728.1
Bacillus toyonensis strain UTDF19-29B chromosome, complete genome	Bacillus toyonensis	100%	4.00E-44	100	5240743	CP081872.1
Bacillus anthracis strain ChBA30D chromosome, complete genome	Bacillus anthracis	100%	4.00E-44	100	5228205	CP091767.1
Bacillus anthracis strain PNO2D1 chromosome, complete genome	Bacillus anthracis	100%	4.00E-44	100	5229500	CP091765.1
Bacillus anthracis strain PNO2 chromosome, complete genome	Bacillus anthracis	100%	4.00E-44	100	5229474	CP091762.1
Bacillus cereus strain FS2 chromosome	Bacillus cereus	100%	4.00E-44	100	5231857	CP090940.1
Bacillus thuringiensis strain ZZQ-130 chromosome	Bacillus thuringiensis	100%	4.00E-44	100	5383145	CP089521.1

Bacillus cereus strain NR1 chromosome, complete genome	Bacillus cereus	100%	4.00E-44	100	5347477	CP090421.1
Bacillus cereus strain CPT56D-587-MTF chromosome, complete genome	Bacillus cereus	100%	4.00E-44	100	5668734	CP090081.1
Bacillus anthracis strain BF5 chromosome, complete genome	Bacillus anthracis	100%	4.00E-44	100	5213322	CP089993.1
Bacillus cereus 30090 DNA, complete genome	Bacillus cereus	100%	4.00E-44	100	5156311	AP023005.1
Bacillus cereus 30077 DNA, complete genome	Bacillus cereus	100%	4.00E-44	100	5358892	AP023000.1
Bacillus cereus 30075 DNA, complete genome	Bacillus cereus	100%	4.00E-44	100	5759674	AP022994.1
Bacillus cereus 30052 DNA, complete genome	Bacillus cereus	100%	4.00E-44	100	5269264	AP022986.1
Bacillus cereus 30043 DNA, complete genome	Bacillus cereus	100%	4.00E-44	100	5356715	AP022978.1
Bacillus cereus 30040 DNA, complete genome	Bacillus cereus	100%	4.00E-44	100	5357266	AP022975.1
Bacillus cereus J75 DNA, complete genome	Bacillus cereus	100%	4.00E-44	100	5329774	AP022970.1
Bacillus cereus J62 DNA, complete genome	Bacillus cereus	100%	4.00E-44	100	5459613	AP022964.1
Bacillus cereus J51 DNA, complete genome	Bacillus cereus	100%	4.00E-44	100	5350395	AP022956.1
Bacillus cereus J39 DNA, complete genome	Bacillus cereus	100%	4.00E-44	100	5344852	AP022952.1
Bacillus cereus J10 DNA, complete genome	Bacillus cereus	100%	4.00E-44	100	5342104	AP022946.1
Bacillus cereus J7 DNA, complete genome	Bacillus cereus	100%	4.00E-44	100	5355432	AP022934.1
Bacillus cereus J2 DNA, complete genome	Bacillus cereus	100%	4.00E-44	100	5241522	AP022927.1
Bacillus cereus J1 DNA, complete genome	Bacillus cereus	100%	4.00E-44	100	5348515	AP022921.1
Bacillus cereus MRY14-0105 DNA, complete genome	Bacillus cereus	100%	4.00E-44	100	5375661	AP022915.1
Bacillus cereus MRY14-0100 DNA, complete genome	Bacillus cereus	100%	4.00E-44	100	5203820	AP022907.1
Bacillus cereus MRY14-0079 DNA, complete genome	Bacillus cereus	100%	4.00E-44	100	5367909	AP022903.1
Bacillus cereus MRY14-0075 DNA, complete genome	Bacillus cereus	100%	4.00E-44	100	5650815	AP022894.1
Bacillus cereus MRY14-0074 DNA, complete genome	Bacillus cereus	100%	4.00E-44	100	5250560	AP022886.1

Bacillus cereus MRY14-0060 DNA, complete genome	Bacillus cereus	100%	4.00E-44	100	5210826	AP022877.1
Bacillus cereus MRY14-0057 DNA, complete genome	Bacillus cereus	100%	4.00E-44	100	5374664	AP022874.1
Bacillus cereus MRY14-0045 DNA, complete genome	Bacillus cereus	100%	4.00E-44	100	5373323	AP022857.1
Bacillus toyonensis strain HA0190 chromosome, complete genome	Bacillus toyonensis	100%	4.00E-44	100	5235892	CP087103.1
Bacillus sp. SD-4 chromosome	Bacillus sp. SD-4	100%	4.00E-44	100	5571932	CP083987.1
Bacillus sp. CRB-7 chromosome, complete genome	Bacillus sp. CRB-7	100%	4.00E-44	100	5254393	CP083749.1
Bacillus anthracis strain P04210076 chromosome, complete genome	Bacillus anthracis	100%	4.00E-44	100	5226685	CP081175.1
Bacillus mycoides strain JAS 83/3 chromosome, complete genome	Bacillus mycoides	100%	4.00E-44	100	5390204	CP067053.1
Bacillus thuringiensis strain GR007 chromosome, complete genome	Bacillus thuringiensis	100%	4.00E-44	100	5659016	CP076539.1
Bacillus cereus strain CF4-51 chromosome, complete genome	Bacillus cereus	100%	4.00E-44	100	5346135	CP063158.1
Bacillus mycoides strain JAS06/1 chromosome, complete genome	Bacillus mycoides	100%	4.00E-44	100	5352400	CP072061.1
Bacillus mycoides strain JAS12/5 chromosome, complete genome	Bacillus mycoides	100%	4.00E-44	100	5265397	CP072057.1
Bacillus mycoides strain JAS85/1 chromosome, complete genome	Bacillus mycoides	100%	4.00E-44	100	5339421	CP072055.1
Bacillus mycoides strain BPN 08/1 chromosome, complete genome	Bacillus mycoides	100%	4.00E-44	100	5281449	CP066847.1
Bacillus mycoides strain JAS94/5 chromosome, complete genome	Bacillus mycoides	100%	4.00E-44	100	5275222	CP036145.1
Bacillus mycoides strain JAS85/1 chromosome, complete genome	Bacillus mycoides	100%	4.00E-44	100	5202602	CP036137.1
Bacillus mycoides strain JAS635 chromosome, complete genome	Bacillus mycoides	100%	4.00E-44	100	5473381	CP036121.1
Bacillus mycoides strain JAS481 chromosome, complete genome	Bacillus mycoides	100%	4.00E-44	100	5296574	CP036117.1
Bacillus toyonensis strain JAS411 chromosome, complete genome	Bacillus toyonensis	100%	4.00E-44	100	5361187	CP036111.1
Bacillus mycoides strain JAS391 chromosome, complete genome	Bacillus mycoides	100%	4.00E-44	100	5235951	CP036102.1
Bacillus mycoides strain JAS23/1 chromosome, complete genome	Bacillus mycoides	100%	4.00E-44	100	5313017	CP036099.1
Bacillus toyonensis strain JAS22/1 chromosome, complete genome	Bacillus toyonensis	100%	4.00E-44	100	5307379	CP036094.1

Bacillus mycoides strain JAS15/1 chromosome, complete genome	Bacillus mycoides	100%	4.00E-44	100	5291025	CP071811.1
Bacillus toyonensis strain JAS13/1 chromosome, complete genome	Bacillus toyonensis	100%	4.00E-44	100	5360709	CP036090.1
Bacillus mycoides strain JAS12/5 chromosome, complete genome	Bacillus mycoides	100%	4.00E-44	100	5233313	CP036085.1
Bacillus mycoides strain JAS1004 chromosome, complete genome	Bacillus mycoides	100%	4.00E-44	100	5407642	CP036084.1
Bacillus mycoides strain JAS06/3 chromosome, complete genome	Bacillus mycoides	100%	4.00E-44	100	5348830	CP036064.1
Bacillus mycoides strain JAS06/1 chromosome, complete genome	Bacillus mycoides	100%	4.00E-44	100	5361170	CP036057.1
Bacillus toyonensis strain JAS03/3 chromosome, complete genome	Bacillus toyonensis	100%	4.00E-44	100	5281277	CP036052.1
Bacillus mycoides strain BPN601 chromosome, complete genome	Bacillus mycoides	100%	4.00E-44	100	5245175	CP036046.1
Bacillus mycoides strain BPN58/4 chromosome, complete genome	Bacillus mycoides	100%	4.00E-44	100	5248764	CP036043.1
Bacillus mycoides strain BPN573 chromosome, complete genome	Bacillus mycoides	100%	4.00E-44	100	5281925	CP036040.1
Bacillus mycoides strain BPN52/2 chromosome, complete genome	Bacillus mycoides	100%	4.00E-44	100	5266779	CP036023.1
Bacillus mycoides strain BPN51/1 chromosome, complete genome	Bacillus mycoides	100%	4.00E-44	100	5281951	CP036017.1
Bacillus toyonensis strain BPN45/4 chromosome, complete genome	Bacillus toyonensis	100%	4.00E-44	100	5446351	CP036014.1
Bacillus mycoides strain BPN37/2 chromosome, complete genome	Bacillus mycoides	100%	4.00E-44	100	5490503	CP036004.1

Table S3 Bacterial strains, plasmids and primers used in this study.

Strain/ Plasmid /Primer	Description	purposes	Source (Reference)
**Stains**			
BMB171	B. thuringiensis strain BMB171; an acrystalliferous mutant strain; high transformation frequency		our lab
BMB171△*nagR2*	BMB171 deleted of *nagR2*		this study
BMB171(pHT1K-*nupR*-p14)	BMB171 containing plasmid pHT1K-*nupR*-p14	β-galactosidase assays	this study
BMB171(pHT1K-*nupR*-p14CT)	BMB171 containing plasmid pHT1K-*nupR*-p14CT	β-galactosidase assays	this study
Δ*nagR2* (pHT1K-*nupR*-p14)	ΔnagR2 containing plasmid pHT1K-*nupR*-p14	β-galactosidase assays	this study
△*nagR2*（pKSV7-P*nupR*-*nup*R）	ΔnagR2 containing plasmid pKSV7-P*nupR*-*nupR*	β-galactosidase assays	this study
BMB171(pHT1K-*nupR*-p14-CcpAmu)	BMB171 containing plasmid pHT1K-*nupR*-p14-CcpAmu	β-galactosidase assays	this study
BMB171(pHT1K-*nupR*-p14-PurRmu)	BMB171 containing plasmid pHT1K-*nupR*-p14-PurRmu	β-galactosidase assays	this study
BMB171(pHT1K-*nupR*-p14-ComKmu)	BMB171 containing plasmid pHT1K-*nupR*-p14-ComKmu	β-galactosidase assays	this study
E. coli DH5α	F- Φ80*lacZ*ΔM15 Δ(*lacZYA-argF*) U169 *rec*A1 *end*A1 *hsd*R17(rk$^-$, mk$^+$) *pho*A *sup*E44 *thi*-1 *gyr*A96 *rel*A1 λ-		
E. coli BL21(DE3)	F2 dcm ompT hsdS (rB 2 mB 2) gall(DE3)		
BL21(pET*nagR2*)	BL21(DE3) with pET*nagR2* plasmid	protain purification	this study
BL21(pET*purR*)	BL21(DE3) with pET*purR* plasmid	protain purification	this study
BL21(pET*comK*)	BL21(DE3) with pET*comK* plasmid	protain purification	this study
BL21-pET-CcpA	BL21(DE3) with pET*ccpA* plasmid	protain purification	our lab
**Plasmid**			
pHT1K-lacZ	B. thuringiensis-*E. coli* shuttle plasmid; Amp^R Erm^R, pHT1K vector harboring the promoter-less *lacZ* gene, transformed into BMB171 and used for β-galactosidase activity	β-galactosidase assays	
pHT1K-*nupR*-p14	lacZ with the promoter of *nupR*-p14 in Nco I and Bam HI sites of pHT1K	β-galactosidase assays	this study
pHT1K-*nupR*-p14CT	compared to pHT1K-*nupR*-p14, a base "C" was mutated to "T"	β-galactosidase assays	this study
pRP1028	*B. thuringiensis-E. coli* shuttle plasmid; Amp^R Erm^R; containing *turbo-rfp* gene and an I-*Sce* I recognition site	gene-knockout	
pRP1028-*nagR2*LR	pRP1028 with the upstream and downstream regions of *nagR2*, intermediate vector in gene-knockout experiments	gene-knockout	this study
pHT1K-*nupR*-p14-CcpAmu	lacZ with the promoter of *nupR*-p14 with CcpA binding box mutant in Nco I and Bam HI sites of pHT1K		
pHT1K-*nupR*-p14-	lacZ with the promoter of *nupR*-p14 with PurR binding box		

PurRmu	mutant in Nco I and Bam HI sites of pHT1K		
pHT1K-*nupR*-p14-	lacZ with the promoter of *nupR*-p14 with ComK binding box		
ComKmu	mutant in Nco I and Bam HI sites of pHT1K		
pET28a(+)	Expression vector, Amp^r	protain purification	
pET*nagR2*	pET28a(+) containing *nagR2* gene, Ampr	protain purification	this study
pET*purR*	pET28a(+) containing *purR* gene, Amp^r	protain purification	this study
pET*comK*	pET28a(+) containing *comK* gene, Amp^r	protain purification	this study
pKSV7	Amp^r Em^r Cm^r; Bacillus-E. coli shuttle vector, temp sensitive	gene complement	
pKSV7-P*nupR*-*nupR*	pKSV7 containing *nupR* promoter and nagR2 CDS	gene complement	this study
**Primers**			
nupR-p14F	TAATACGATCACTATAGGGCCATGGCCGAGAGAAGTACTCATTAATG	β-galactosidase assays	this study
nupR-p14R	TAGTGTATCAACAAGCTGGGGATCCATGTAAGACT TCTTTTCTCTTTTTTCAACTTAC	β-galactosidase assays	this study
nupR-p14CTuF	AGATCTAATACGATCACTATAGGGCCATGGCCGAGAGAAGTACTCATTAATG	β-galactosidase assays	this study
nupR-p14CTuR	ACTTTTCCCCTCATAATCTAGCAATTCATGGT	β-galactosidase assays	this study
nupR-p14CTdF	ACCATGAATTGCTAGAtTATGAGGGGAAAAGT	β-galactosidase assays	this study
nupR-p14CTdR	CATTAGTGTATCAACAAGCTGGGGATCCTGACATAAGACTCCCCCGCATTTC	β-galactosidase assays	this study
nagR2-F	TGGTGGTGGTGGTGCTCGAGCATACGCTTTCTTAATAC	protain purification	this study
nagR2-R	AGAAGGAGATATACCATGGGCATGCGGGGGAGTCTTATG	protain purification	this study
purR-F	GTGGTGGTGGTGGTGGTGCTCGAGTTCCGCCTCTACAAGCCCTT	protain purification	this study
purR-R	GAAGGAGATATACCATGGGCATGAAAATTAGAAGAAGTAC	protain purification	this study
comK-F	TGGTGGTGGTGGTGCTCGAGATGTAGTAAATGTAACAAGGGGTTT	protain purification	this study
comK-R	GAAGGAGATATACCATGGGCATGAATGATGAAAATAACATTATT	protain purification	this study
*nagR2*L-F	TTTAAGGCCAACGAGGCCGCAAGATGTTCCTGATACGAAGC	gene-knockout	this study
*nagR2*L-R	CTGTTTCAGGCTCACATTCTGTCGTTCTTTATAAGCCCCATC	gene-knockout	this study
*nagR2*R-F	GCTTATAAAGAACGACAGAATGTGAGCCTGAAACAGCACTG	gene-knockout	this study
*nagR2*R-R	CAAGGCCTTATTGGCCTACACCGTTACCTGTACCACCAGCA	gene-knockout	this study
nagR21	GGACGAGTTAGCAGATTTAATGATGG	verification of gene-knockout	this study
*nagR2*2	CATACCTTCTTGATGTTGGTCACGG	verification of gene-knockout	this study
*nupR*com-F	CCGGAATTCGGATGTAAAGGATCTTTGTGCCGAACTTT	gene complement	this study
*nupR*com-R	CTAGTCTAGACTACATACGCTTTCTTAATACGTGGAAGC	gene complement	this study
16SF(QP)	CCGCGGTAATACGTAGGTG	qRT PCR	this study
16SR(QP)	TTTCCAATGACCCTCCACG	qRT PCR	this study
bgaBF(QP)	TGGAGCAAGATCGAACCGTC	qRT PCR	this study
bgaBR(QP)	AGCTGGAGTAGTTGCAGTCG	qRT PCR	this study
cggRF(QP)	AAAAGACCGTCTGACAGCGA	qRT PCR	this study
cggRR(QP)	CGACGCCTTCTCCAATTCCA	qRT PCR	this study
pbuGF(QP)	TACGGGGATGTGTTTGGAGC	qRT PCR	this study

pbuGR(QP)	AGCACGTGGGAATTTACGGT	qRT PCR	this study
nagEF(QP)	TTCGTTTAGGGCAACCAGAT	qRT PCR	this study
nagER(QP)	CATCCACTGAAAGACCGATT	qRT PCR	this study
narGF(QP)	ACGAACGGTCGCTCAATGAT	qRT PCR	this study
narGR(QP)	CTTGTGCTCCGACGAGAAGT	qRT PCR	this study
hppDF(QP)	CGTTTCGTTGTGTCTGGAGC	qRT PCR	this study
hppDR(QP)	CACGCCATCACCATGAGTCT	qRT PCR	this study
queGF(QP)	TCAGAGCGTGCTGGTATTGG	qRT PCR	this study
queGR(QP)	GCTTATCTGGCGGGAATGGA	qRT PCR	this study
mdlAF(QP)	CGCTCGTTGGAGAGAAAGGT	qRT PCR	this study
mdlAR(QP)	AACAGCTCGTGCAATCGAGA	qRT PCR	this study
ykfCF(QP)	ATGAAATGTGGTGGCATACG	qRT PCR	this study
ykfCR(QP)	CGGAAAGTGAAGACTCCATCA	qRT PCR	this study
pipF(QP)	CTGGTGATTACTTTGCGGAT	qRT PCR	this study
pipR(QP)	ATGCTTCTCGTATGGCTTCC	qRT PCR	this study
P*nagR2*-F	GGCAACCATGAATTGCTAGACTATG	EMSA	this study
P*nagR2*-R	CGCATTTCAATTATTCGACCTCTGATG	EMSA	this study
P*nupC*1-F	CGGACGATGTTACCTCATATATACTTG	EMSA	this study
P*nupC*1-R	CCGATAATGCTCATTACATATTGC	EMSA	this study
P*nupC*2-F	GGGCTATTTTCATGGTAAATTTAC	EMSA	this study
P*nupC*2-R	ATATGTGAATCCTCCACTTCTCCC	EMSA	this study
P*nupC*3-F	GGTATAATGAAAAGGGCATCAAGTG	EMSA	this study
P*nupC*3-R	CCTACAAGGAACATAACAAACTTC	EMSA	this study
P*nupC*4-F	GCTATGAGTTCAACAATTACTCCC	EMSA	this study
P*nupC*4-R	GATTCATGCAGTGTATGCTCCTTCC	EMSA	this study
P*baci*-F	TGCATACTTACTCACCATCCCAT	EMSA	this study
P*baci*-R	CTATAATTCATCTATAACACTCC	EMSA	this study
P*had*-F	AACTTCCATTATTGCACTCTACC	EMSA	this study
P*had*-R	GGTGAAATAGTATGGTCATCACG	EMSA	this study
P*ldh*-F	CCATGCCATTTCCCCTTTTACAAA	EMSA	this study
P*ldh*-R	CTAATACAACACGGTTAATACC	EMSA	this study
3461_F	AGAGTTTGATTTAGCGAAAGAACTTGGC	RT-PCR	this study
3461_R	ACAATACACAACAGGTTCCCCATCT	RT-PCR	this study
61_60_F	GTGAGCCTGAAACAGCACTGCT	RT-PCR	this study
61_60_R	GCCTTATCCGAGTTACCACATGC	RT-PCR	this study
3460_F	GCATGTGGTAACTCGGATAAGGC	RT-PCR	this study
3460_R	CGGTGCATCTACAACAGTATCTACG	RT-PCR	this study
60_59_F	GCCGGTGAAATTAAAGTGCCAG	RT-PCR	this study
60_59_R	TTCCCTGCACCATTTTCTCCAA	RT-PCR	this study
3459_F	TTGGAGAAAATGGTGCAGGGAA	RT-PCR	this study
3459_R	CTCAACACGCTGCTGCATACC	RT-PCR	this study
59_58_F	TATTAATTGCGGCACAGCCAAC	RT-PCR	this study
59_58_R	AGCATTACAATCGCTCCTACAAGT	RT-PCR	this study
3458_F	GCATTGGTGGGCGGAATTTGG	RT-PCR	this study

3458_R	CAATAATTCCCCAGTGTAGACGT	RT-PCR	this study
58_57_F	GGAGTAGGATTTGATGGGATTGC	RT-PCR	this study
58_57_R	TTACAACACCCGATCGTTCACT	RT-PCR	this study
3457_F	TGGAATTGGTGGAGCGATTTT	RT-PCR	this study
3457_R	ACCTACAAAACCGACAAGCGCTA	RT-PCR	this study
nagR2raceF1	CCGAGAGAAGTACTCATTAATG	5'RACE	this study
nagR2raceR2	TCCTCATTTCGAGAAAAAGTTCGGCAC	5'RACE	this study
nagR2raceR1	ATGTAAGACTTCTTTTCTCTTTTT	5'RACE	this study
primer8	GACCACGCGTATCGATGTCGACTTTTTTTTTTTTTTTTV	5'RACE	this study
ccpAf-emsa	ACCGAGAGAAGTACTCATTAATGATG	EMSA	this study
ccpAr-emsa	ATGTAAGACTTCTTTTCTCTTTTTAATCTTACCACTTGCTCTCATACG	EMSA	this study
comkf-emsa	CGAAATGAGGAATGTTTATGGTGC	EMSA	this study
comkr-emsa	ATGTAAGACTTCTTTTCTCTTTTT	EMSA	this study
comkf1	AATACGATCACTATAGGGCCATGGAGTAGGTCCGTATGAAGGGA	β-galactosidase assays	this study
comkf2	TGCAATAAAGAGTGAAAATTTTAGGGGAAAAAATT	β-galactosidase assays	this study
comkr1	AATTTTTTCCCCTAAAATTTTCACTCTTTATTGCA	β-galactosidase assays	this study
comkr2	TGTATCAACAAGCTGGGGATCCATGTAAGACTTCTTTTCTCTTTTT	β-galactosidase assays	this study
purrf1	CGATCACTATAGGGCCATGGAGTAGGTCCGTATGAAGGGA	β-galactosidase assays	this study
purrf2	GTATGTTTTATGTGGATGTTTTGTATCTAATGCAGAAA	β-galactosidase assays	this study
purrr1	TTTCTGCATTAGATACAAAACATCCACATAAAACATAC	β-galactosidase assays	this study
purrf3	TTTCTCGAAATGAGGAGTGTCCATGGAGCAAAGTTTT	β-galactosidase assays	this study
purrr3	TGTATCAACAAGCTGGGGATCCATGTAAGACTTCTTTTCTCTTTTT	β-galactosidase assays	this study
purrr2	AAAACTTTGCTCCATGGACACTCCTCATTTCGAGAAA	β-galactosidase assays	this study
ccpaf1	AATACGATCACTATAGGGCCATGGAGTAGGTCCGTATGAAGGGAGTA	β-galactosidase assays	this study
ccpAr1	CACCCCTAAATATAAATCCGCTATTTATCGCTGAGTAT	β-galactosidase assays	this study
ccpAf2	ATACTCAGCGATAAATAGCGGATTTATATTTAGGGGTG	β-galactosidase assays	this study
ccpAr2	TGTATCAACAAGCTGGGGATCCATGTAAGACTTCTTTTCTCTTTTT	β-galactosidase assays	this study
